# Heritable Epigenomic Modifications Influence Stress Resilience and Rapid Adaptations in the Brown Planthopper (*Nilaparvata lugens*)

**DOI:** 10.3390/ijms23158728

**Published:** 2022-08-05

**Authors:** Ayushi Gupta, Suresh Nair

**Affiliations:** Plant-Insect Interaction Group, International Centre for Genetic Engineering and Biotechnology (ICGEB), Aruna Asaf Ali Marg, New Delhi 110067, India

**Keywords:** insect epigenetics, DNA methylation, *Nilaparvata lugens*, adaptive stress response, genetic plasticity, plant-insect interactions

## Abstract

DNA methylation in insects is integral to cellular differentiation, development, gene regulation, genome integrity, and phenotypic plasticity. However, its evolutionary potential and involvement in facilitating rapid adaptations in insects are enigmatic. Moreover, our understanding of these mechanisms is limited to a few insect species, of which none are pests of crops. Hence, we studied methylation patterns in the brown planthopper (BPH), a major rice pest, under pesticide and nutritional stress, across its life stages. Moreover, as the inheritance of epigenetic changes is fundamentally essential for acclimation, adaptability, and evolution, we determined the heritability and persistence of stress-induced methylation marks in BPH across generations. Our results revealed that DNA methylation pattern(s) in BPH varies/vary with environmental cues and is/are insect life-stage specific. Further, our findings provide novel insights into the heritability of stress-induced methylation marks in BPH. However, it was observed that, though heritable, these marks eventually fade in the absence of the stressors, thereby suggesting the existence of fitness cost(s) associated with the maintenance of the stressed epigenotype. Furthermore, we demonstrate how 5-azacytidine-mediated disruption of BPH methylome influences expression levels of stress-responsive genes and, thereby, highlight demethylation/methylation as a phenomenon underlying stress resilience of BPH.

## 1. Introduction

Genome plasticity is a key attribute underlying the ability of insects to produce a wide range of phenotypes in response to environmental cues, enabling their remarkable ecological success [1,2]. Rapid alterations in insects’ biochemical, physiological, and metabolic processes and shifts in their developmental trajectory are hallmarks of their quick response to any environmental change favouring their survival and reproduction [3,4,5]. Several studies have demonstrated that these responses are the outcome of epigenetic processes, such as DNA methylation, that mediate insect polyphenisms and developmental plasticity [6,7]. 

DNA methylation is regarded as one of the most faithfully heritable forms of epigenetic modification brought about by the action of DNA methyltransferases [8,9]. Although invertebrates display negligible to intermediate amounts of DNA methylation [10,11], mainly confined to gene bodies [12,13], its manipulation is known to disrupt normal patterns of plasticity, such as the development of caste in social insects [14,15]. Recent advancements focusing on understanding phenotypic and genetic plasticity through the characterisation of the various physiological and molecular processes provide important cues regarding the involvement of DNA methylation in conferring adaptive advantages to insects. Studies have shown that alteration in DNA methylation patterns in insects can cause significant changes in the gene expression profile, lead to the generation of alternate splice forms (mRNA variants), exon shuffling events and chromatin remodelling, and impact genome structure and organisation [16]. All these changes can ultimately influence and redirect cellular differentiation and predetermined developmental programs in insects in response to external stimuli. 

Epigenetic modifications, including DNA methylation, involve a vast array of chemical changes as a part of epigenetic molecular mechanisms (EMMs) operating in an insect. EMMs are reported in several insect species, where these processes are shown to be actively involved in regulating and stabilizing critical functions and events in the insect’s life. Further, it has been speculated that EMMs, in several phytophagous insects, contribute to their resilience to various environmental stresses posed by pesticides, temperature, etc. [17,18]. However, we believe that in addition to providing resistance/tolerance capacity to various abiotic stresses, they can also help insects overcome host plant resistance.

In this regard, although recent studies have indicated the involvement of epigenetic processes in insect’s fitness and survival [19,20], conclusive evidence for DNA methylation as a mediator of the rapid adaptive stress response(s) remains/remain elusive. Besides, we are yet to fully correlate methylation patterns with phenotypic plasticity observed in wild insect populations. Similarly, despite the common belief that methylation probably underlies rapid adaptive stress response in insects, we know very little about whether differential methylation is associated with an insect’s performance under any particular stress. For this, we first need to study changes in DNA methylation under control conditions to subsequently correlate them with the observed insect phenotype. With this view in mind, we investigated DNA methylation patterns in a laboratory-reared population of *Nilaparvata lugens* [21], also known as brown planthopper (Hemiptera; Delphacidae; BPH), in response to two major physiological conditions, i.e., pesticide and nutritional stress. BPH, a monophagous sap-sucking insect, has emerged as one of the major pests of rice over the past few years and is ubiquitously found in all the major rice-growing regions. Its infestation results in a massive reduction in the total crop yields, thereby causing considerable losses to the farmers. Conventional pest-management strategies are proving unsuccessful, with populations of BPH rapidly building resistance to most pesticides and, thereby, rendering them ineffective. Several rice varieties currently deployed in the field are highly susceptible to BPH, due to the ability of the latter to rapidly break down host plant resistance. Therefore, we deemed BPH a good model for investigating mechanisms underlying the rapid adaptive nature of insect pests. 

Interestingly, it has been observed that insects exposed to hostile environments (i.e., abiotic and biotic stress) often prime their future generations to better tolerate/resist that stress [22]. This further supports the idea of EMMs being involved in this process, as epigenetic modifications are mitotically and meiotically heritable across cell divisions and generations [18]. DNA methylation can well explain instances of trans-generational plasticity, wherein the phenotype induced in the parental generation is observed in their offspring. Inherited methylation patterns provide a possible explanation for the wide range of phenotypic variations exhibited by resistance traits, i.e., why individuals in a population show variable levels (slow to fast) of response to a novel or rapidly changing environment. 

Over the past few years, the idea that ‘genetic code’ is the sole factor involved in biological inheritance has been challenged by studies reporting the existence of transgenerational epigenetic inheritance [23]. ‘Epigenetic inheritance’ refers to the transfer of information across generations without involving any nucleotide change in the DNA sequence. Owing to the transfer of components that regulate how DNA is read and expressed, environmentally triggered phenotypes can persist in a population for several generations [24]. However, despite several reports from vertebrates, this phenomenon has only been recently discovered in invertebrates. Although transgenerational epigenetic inheritance has now been experimentally reported at functional and evolutionary levels in some insect models, it is difficult to comprehend the broader implications from available studies. While inclusion/exclusion of DNA methylation marks could profoundly influence gene structure and function and determine insect adaptation to environmental fluctuations, their role as carriers of heritable information is still enigmatic. Moreover, the evolutionary significance of epigenetic inheritance in arthropods remains controversial and inconsistent across insect orders. 

Previous studies demonstrated that invertebrate taxa, such as dipterans and insects that are social and solitary, exhibit negligible or minimal epigenetic reprogramming [25]. However, and in contrast, several recent reports suggest that heritable epigenetic mechanisms have influenced the evolution of bees, wasps, and ants [26]. The study of DNA methylation in *Nasonia* wasps revealed the existence of DNA methylation patterns in F1 generation corresponding to that found in the parental genome, thereby suggesting epigenetic inheritance [27]. Similarly, other studies have shown that gene expression patterns exhibit some commonality between the parental generation and their offspring [28]. However, we believe it is crucial to extend such investigations to other insect species to obtain a better understanding of the inheritance of epigenetic marks in invertebrates, which is currently limited to few insect models and none, as far as we are aware, for insects that are important pests of major crop plants. With this view in mind, and in addition to investigating DNA methylation patterns in BPH under different physiological conditions, we also studied the heritability of stress-induced methylation marks across generations. 

Epigenetic inheritance can indeed be vital for insect adaptations to novel environments, especially when the level of genetic variability present within that insect is lacking or minimal. Under such circumstances, epigenetically-induced variability can not only enable insects to survive the fluctuating environments but also allow insect populations to quickly respond to the change, thus favouring their adaptation and evolution [29]. In addition, methylated sites in the genome are regarded as ‘mutation hotspots’ for cytosine to thymine conversion [30], hence, the source of genetic variation, which can sometimes be adaptive. Moreover, it has been observed that the rate of methylation and demethylation (i.e., the epimutation rate) is generally higher than the genetic mutation rate [31], which acts as the additional source of heritable variations that can influence insect adaptation. However, initiation of epigenetic reprogramming and subsequent maintenance can demand huge energy investments and come with high metabolic costs [32]. Some findings suggest that as epigenetic inheritance is a costly phenomenon, its occurrence requires strong selection pressure [33]. It can sometimes have repercussions in terms of levying fitness costs upon the offspring that might have to be borne by them for several generations. 

With the rapidly expanding field of epigenetic studies addressing insects, we now have data from a few insect species indicating that environmentally induced DNA methylation marks can last for two to four generations, or sometimes even longer, in an insect population [34,35,36]. However, the information regarding the maintenance of stress-induced methylation marks, especially in the absence of the stressor, is scarce. Therefore, in addition to demonstrating epigenetic reprogramming and its inheritance, we also investigated the duration of persistence of the stress-induced epigenetic marks in BPH populations to determine the relevance of taking fitness costs into account while studying an organism’s life-history trade-offs and dynamics across generations. 

Pesticides and resistant host varieties are the two major stressors phytophagous insects face during their lifetime. Of a wide range of chemicals used in modern-day agriculture, imidacloprid is one of the most extensively used pesticides for controlling BPH outbreaks [37]. Therefore, we chose to analyze the impact of imidacloprid (a neonicotinoid pesticide) on the methylation status of BPH. Additionally, the involvement of DNA methylation in assisting BPH to tolerate resistant rice varieties (i.e., nutritional stress) was also investigated. We studied the ‘epigenotype’ of BPH with regards to a set of selected stress-responsive genes to test whether methylation patterns display a trend in BPH populations upon exposure to pesticide and nutritional stress across its developmental stages. In addition, we screened the first and fourth generations of the insects exposed to these stresses for the presence of methylation marks, similar to those detected in the parental genome. Further, we also assessed insects from the reversal populations (i.e., after they were brought back to favourable growing conditions) to evaluate the time taken for these epigenetic marks to fade away and determine if there is any fitness cost(s) associated with the maintenance of the stressed epigenotypes. 

Furthermore, to confirm and validate the functional relevance of DNA methylation for BPH survival, BPH insects were treated with 5-azacytidine (5-aza; an exclusive DNA methylation inhibitor) and evaluated for perturbations in methylation status and gene expression. Apart from modulating gene expression, methylation is also associated with exon shuffling and alternative splicing [38]. Therefore, to address these aspects, in silico predictions for identifying alternate splice sites within the CpG islands (corresponding to the selected stress-responsive genes employed in this study) were also performed. 

## 2. Results

### 2.1. BPH Populations, Insecticide Resistance, and Callose Plug Formation in RH Plants

**Figure 1:**
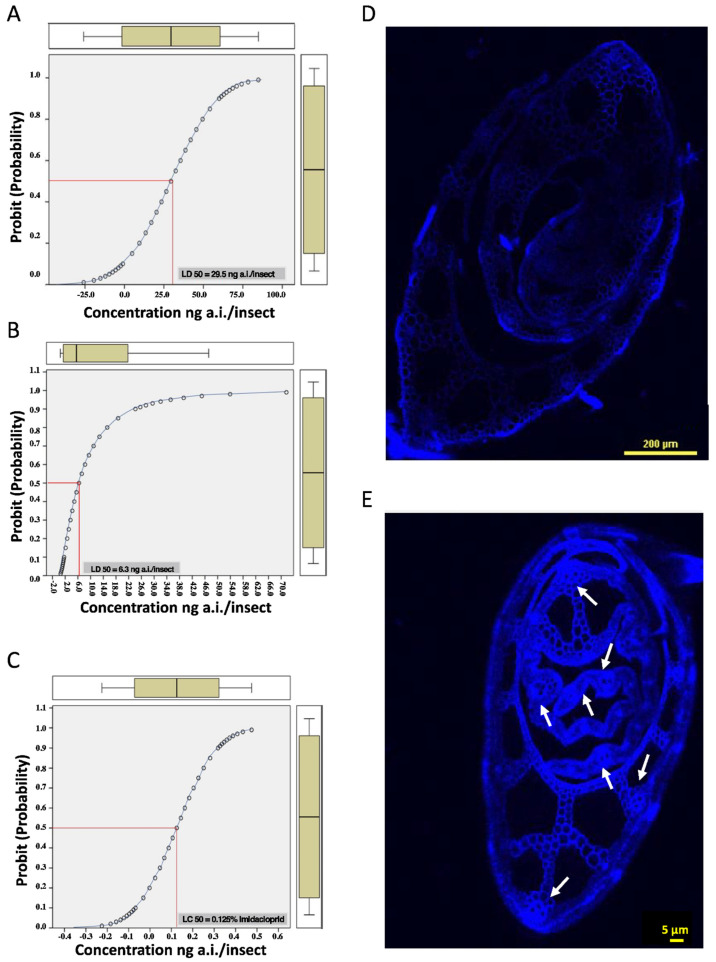
BPH populations subjected to pesticide and nutritional stress. (**A**) Mortality curve, estimated by the dose-response (Probit) analysis, for BPH (adults) 24 h after exposure to imidacloprid. LD_50_ estimate (response vs. amount of active ingredient (a.i.) applied topically per insect) for the field-collected BPH population. (**B**) LD_50_ estimate for the BOD population. (**C**) LC_50_ mortality curve (response vs. concentration expressed as percentage of soluble concentrate (SL) of imidacloprid) for the BOD population of BPH. The respective LD/LC_50_ values of BPH populations are indicated by the red lines. (**D**) Transverse section (TS) of TN1 (susceptible rice variety) stem showing negligible callose deposition on the sieve plates. (**E**) Thick callose deposition (white arrows) observed in Rathu Heenati (RH; resistant rice variety) after BPH infestation.

DNA methylation status is often under- or over-represented due to naturally occurring variations in a DNA sequence. This can pose a serious problem, especially while studying the populations of highly migratory insects, such as BPH. In this regard, and to obtain reliable information on DNA methylation, a highly inbred population of BPH (BOD population) with a LD_50_ value (imidacloprid) approximately five times lower than that of the field population of BPH was generated. While the LD_50_ value of imidacloprid for the field population of BPH was 29.5 ng active ingredient (a.i.) per insect, it was only 6.3 ng a.i./insect for the BOD population (Figure 1A,B). Hence, the BOD population was considered susceptible and, therefore, used as the source population to obtain stress-exposed BPH populations for methylation analyses. The dose (concentration) of imidacloprid required to induce a resistance/tolerance response in the BOD population, as determined by LC_50_ analysis, was 0.125% soluble concentrate of imidacloprid solution (Figure 1C). Further, in the case of nutritional stress, blockage of sieve elements (due to callose deposition) was observed in RH plants post infestation. This indicated BPH feeding and subsequent induction of resistance response(s) in the resistant rice variety (Figure 1D,E). Based on these observations, the insects feeding on RH were considered suitable for methylation analyses. 

### 2.2. Analysis of Global DNA Methylation of the BPH Genome after Exposure to Stress

**Figure 2:**
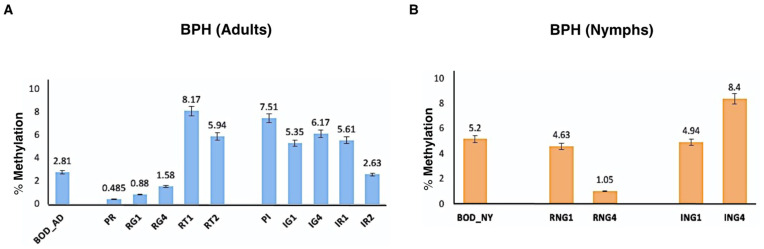
Comparison of global DNA methylation profiles of BPH populations. (**A**) Changes in DNA methylation, upon exposure to pesticide and nutritional stress, in BPH adults (**B**) Response of BPH nymphs with regard to the genome-wide alterations in their DNA methylation levels upon exposure to stress. Error bar represents SD.

Quantitation of 5-methylcytosine in BPH adults and nymphs revealed life-stage specific differences in global DNA methylation levels in the BPH genome. BPH nymphs (BOD_NY) displayed relatively higher levels of methylation (i.e., 5.2%) as compared to adults (BOD_AD), where only 2.81% methylation was detected (Figure 2). Further, the global DNA methylation analysis performed on stress-exposed BPH insects (pesticide and nutritional stress) hinted at the occurrence of epigenetic reprogramming with regard to DNA methylation upon exposure to stress (Figure 2). It was observed that while exposure to imidacloprid resulted in an overall increase in percent methylation (i.e., 7.51% for PI), nutritional stress (RH), in contrast, caused hypomethylation of the BPH genome (i.e., methylation levels dipped to 0.485% for PR) in adults. In addition, it was observed that BPH insects maintained their altered methylation status till the stressors (imidacloprid and RH) persisted (Figure 2).

Further, upon transferring stress-exposed BPH individuals to favourable conditions, BPH insects did not show an immediate but rather a gradual reversal of methylation status. While insects under imidacloprid reverted to their initial methylation state within two generations (i.e., IR2 showed 2.63% methylation), RH insects exhibited a noticeable deviation (increase) in global methylation levels (i.e., RT2 showed 5.94%), as compared to the control insects. Interestingly, nymphs did not show any change in their methylation status upon exposure to stress until 1st generation (i.e., both RNG1 and ING1 had 4.63% and 4.94% methylation, respectively) but showed a dip (i.e., 1.05% for RNG4) under RH stress, and an increase (i.e., 8.4% for ING4) after pesticide exposure by 4th generation.

### 2.3. Targeted Methylation Analysis of Stress-Responsive Genes in BPH 

**Figure 3:**
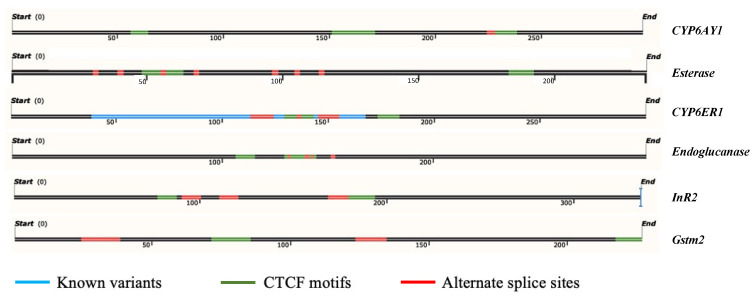
Diagrammatic representation of the location of regulatory motifs detected within the CpG islands, corresponding to stress-responsive genes in BPH. Numbers represent nucleotide position in the sequences.

The present study targeted CpG islands (i.e., regions prone to methylation) of the selected stress-responsive genes (Table S1). These islands were mainly detected in the gene body, and their length ranged from 226–350 bp (Table S2). In addition, in silico analyses predicted the presence of various transcription factor binding sites (TFBSs), CCCTC-binding factor (CTCF) motifs, and alternate splice sites within these CpG islands (Figure 3). The targeted bisulfite sequencing detected methylation marks across these predicted CpG islands for BPH populations (Table S3) at a single base-pair resolution. In this regard, the bisulfite conversion efficiency, based on cytosine conversion rates in the control reaction, was estimated to be >99%. In addition, the DNA recovery post-bisulfite conversion was over 50% (data not shown).

Post-bisulfite treatment, bidirectional sequencing libraries constructed from PCR-amplified fragments corresponding to these CpG islands generated over 9.1 million reads on the Illumina MiSeq Sequencing platform. Of the total reads, >98% were of high quality (Q score cut off >30) and, hence, were retained after base-calling, adapter trimming, and quality filtering for downstream methylation analyses. These high-quality reads accounted for >95% of the total reads obtained for each sample. Next, the bisulfite read pairs were merged and aligned to their respective reference sequences, where reads corresponding to different amplicons were successfully mapped, either to the original top strand (OT), its complementary strand (CTOT; complementary to original top strand), original bottom strand (OB) or its complementary strand (CTOB; complementary to original bottom strand), of the respective reference sequences. The mapping efficiency ranged from 30% to 86% across samples (Table S4).

**Figure 4:**
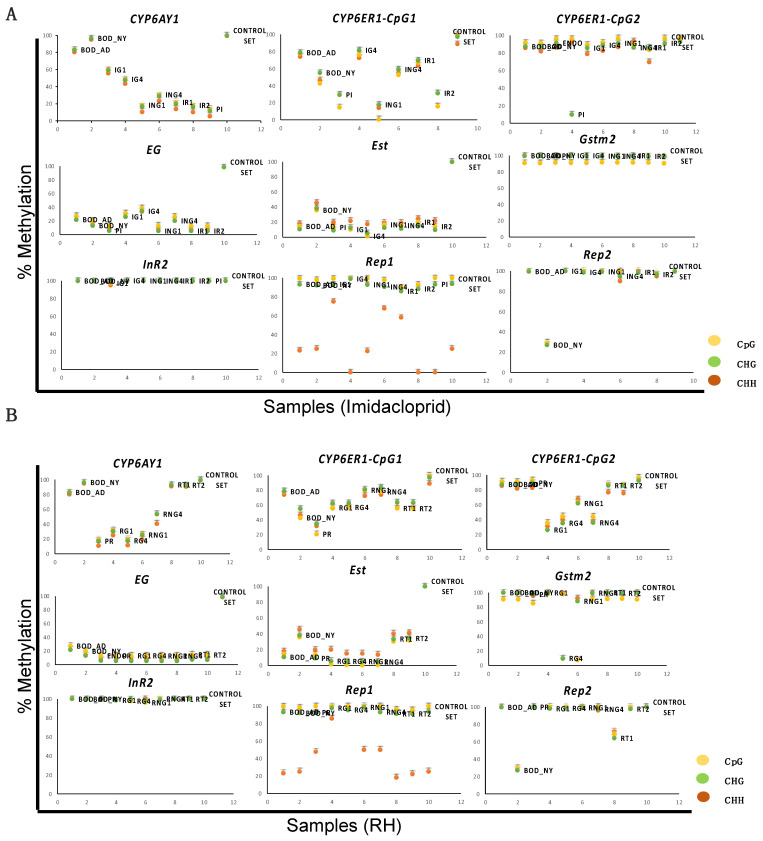
Fluctuations in methylation values of BPH genes (in CG, CHG, and CHH contexts) under stress and after its withdrawal (reversal populations). (**A**) upon exposure to pesticide (imidacloprid). (**B**) under nutritional stress (RH).

**Figure 5:**
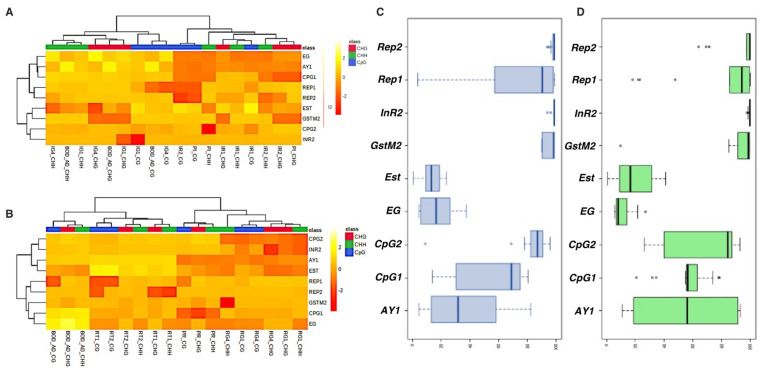
Estimation of variability in methylation levels of genes, across BPH populations (adults), when subjected to stress. (**A**) Heatmap depicting alterations in the methylation status of stress-responsive genes upon exposure to imidacloprid. (**B**) Fluctuations (in methylation of genes) observed under nutritional stress (RH). (**C**) Whisker plots depicting the degree of variability (viz. methylation) exhibited by genes under pesticide stress. (**D**) under nutritional (RH) stress. Mean values, quartiles, standard deviation, and outliers are indicated.

Further, data obtained upon methylation calling (for reads corresponding to each gene across all samples) in all three contexts revealed that the methylation status of BPH varied across genes and between treatments (Table S5, Figure 4). Under pesticide stress, while the methylation levels of *InR2*, *Gstm2*, and *CYP6ER1-CpG2* did not show any considerable change, high alterations were observed for *CYP6AY1*, *CYP6ER1-CpG1*, and *Endoglucanase,* in all three contexts and between life-stages (Figure 4A). However, *Rep1* exhibited variations only in the CHH context, and *Rep2* and *Est* varied only for nymphs (Figure 4A and Figure 5A). Similarly, under nutritional stress, while methylation values for *CYP6AY1*, *CYP6ER1*-*CpG1*, *CYP6ER1*-*CpG2*, and *Est* exhibited variations between stress-exposed BPH and control samples, and also across life stages, *InR2* and *Gstm2* did not show any change (Figure 4B and Figure 5B). Moreover, *Rep1* displayed changes only in the CHH context, and methylation values of *Rep2* differed between adults and nymphs (Figure 4B). Taken together, it was observed that the methylation status of genes in BPH is influenced by external environmental conditions. Besides, the estimation of variability in methylation scores for each gene under pesticide and nutritional stress, using whisker plot analysis, also indicated that the methylation status of genes in the BPH genome is stress-specific (Figure 5C,D).

**Figure 6:**
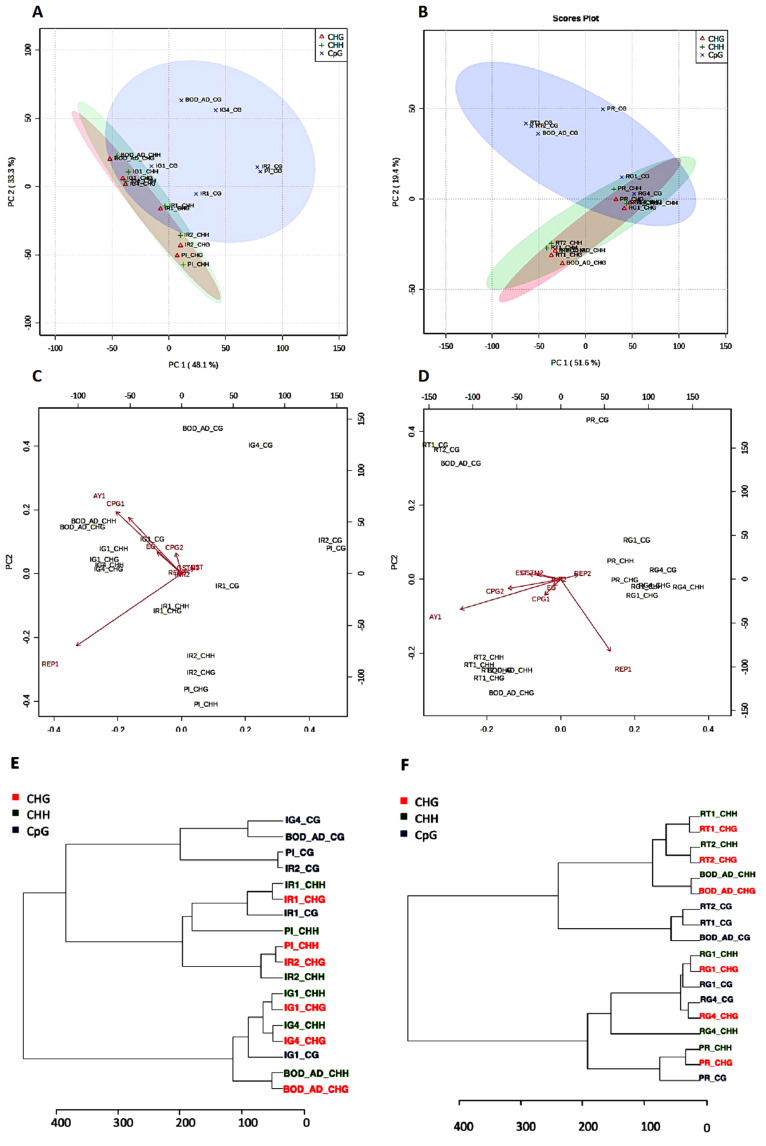
Assessment of the relationship between variables (methylation scores) across BPH populations (adults) exposed to stress and after its withdrawal. (**A**) Principal component analysis (PCA) plot showing distinct methylation patterns in CG and non-CG contexts for pesticide-exposed BPH populations. (**B**) PCA plots for BPH populations fed on RH. (**C**) PCA biplot representing the influence of each gene on the selected PCs under pesticide stress. (**D**) Contribution of each gene to the observed variability under nutritional stress. (**E**) Hierarchical clustering analysis showing the heritability of pesticide-induced changes in DNA methylation, across generations. (**F**) Stress-induced changes displaying immediate reversal in the case of nutritional stress. The reversal populations (RT1 and RT2) resembled the control (BOD population), and, hence, were grouped as a separate clade.

Further, the variance estimation between samples and the assessment of relationship among variables (methylation scores), using PCA analysis, indicated distinct patterns of methylation in CG and non-CG contexts for pesticide and nutritional stresses (Figure 6A–D). Moreover, in accordance with our data on the overall percent methylation, the hierarchical clustering analysis hinted at the heritability of pesticide-induced changes in DNA methylation across generations (Figure 6E). Results showed that reversal samples (IR1 and IR2) exhibited a closer resemblance to PI (i.e., BPH population under pesticide stress) than to the BOD insects (Figure 6E), thereby indicating the transfer of epigenetic information in the form of DNA methylation over generations. However, and in contrast, these changes showed an immediate reversal in the case of nutritional stress (Figure 6F). Here, the reversal populations (RT1 and RT2) resembled the control (BOD population) and, hence, were grouped in a separate clade (Figure 6F). However, before delving deeper into establishing heritability, it was important to confirm the reliability and significance of these findings. With this in mind, the methylation data (across all genes studied) was subjected to statistical analyses (see ‘Methods’ section; Figure S1). As methylation percentage in itself is not reliable with regards to actual coverage of the site (within stress-responsive genes), data of methylation counts for each cytosine, and its respective coverage, were also subjected to statistical analysis (for details, see ‘Methods’ section). The results showed that cytosine sites analysed in the present study differed significantly between treatments, thereby further validating the differences observed in methylation scores between stressed and control BPH populations.

**Figure 7:**
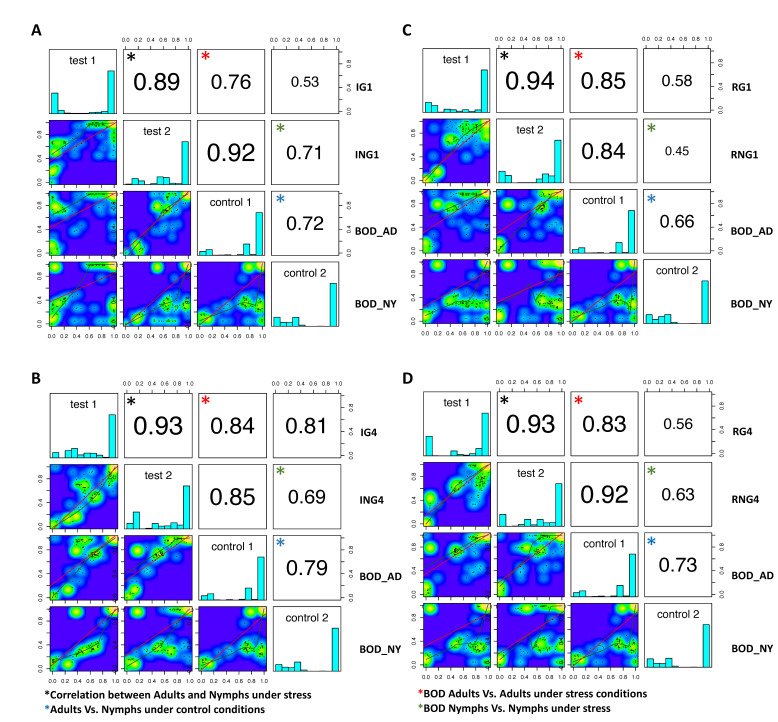
Correlation matrix and scatter plots showing Pearson correlation of CpG methylation (for each cytosine), across BPH populations, analysed in the present study. (**A**) BPH populations under imidacloprid stress (1st generation). (**B**) BPH populations under imidacloprid stress (4th generation). (**C**) BPH populations feeding on RH (1st generation). (**D**) BPH populations feeding on RH (4th generation). Numbers on the upper right corner denote pair-wise Pearson’s correlation scores across samples. The histograms on the diagonal represent CpG methylation level of each sample from 0% to 100% distributed across 10 bins of 10% intervals. Most of the bases have either high or low methylation. The red and green lines on the scatter plots represent linear regression and loess fit, respectively, to model the relationship of differential CpG methylation sites between the compared pairs.

Next, the pairwise Pearson’s correlation scores and scatter plots delineated the impact of the two experimental variables (i.e., life stages and stresses) on the methylation status of BPH genes (Figure 7). This analysis was based on the premise that samples with low correlation scores exhibit higher deviation from the control (BOD population) with regard to their epigenotype. Here, it was observed that although adults and nymphs differed in their methylation status under normal (control) conditions (correlation coefficient 0.66–0.79), they exhibited a similar ‘epigenotype’ (correlation coefficient 0.89–0.94) under stressed conditions (Figure 7). This is in agreement with our earlier findings, based on global DNA methylation analysis, where we observed that BPH displays uniform alterations in methylation status across its different life stages after exposure to stress conditions. However, it is interesting to note that while the changes in methylation at a global scale were evident only after 4 generations of continuous exposure, stress-responsive genes demonstrated a very rapid response, i.e., alteration in their methylation status within 10 days of exposure.

Further, it was observed that nymphs feeding on RH mounted a stronger initial response (1st generation, correlation coefficient 0.45) as compared to adults (correlation coefficient 0.85), which gradually declined by 4th generation (correlation coefficient 0.66). Furthermore, insects under pesticide stress had higher correlation values (i.e., 0.76–0.84 and 0.69–0.71 for adults and nymphs, respectively) than those feeding on RH (correlation coefficient ranged from 0.83–0.85 for adults and 0.45–0.63 for nymphs), thereby indicating that nutritional stress was a stronger stimulus for inducing epigenetic changes in BPH than pesticide stress.

### 2.4. Site-Specific Analysis of the Heritability of Stress-Induced Methylation Marks in BPH

**Figure 8:**
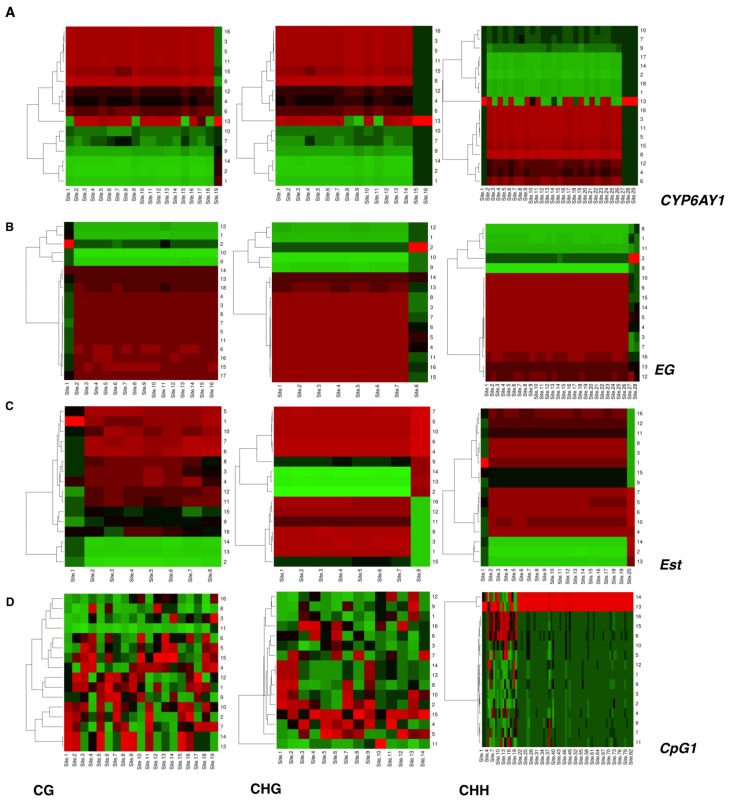

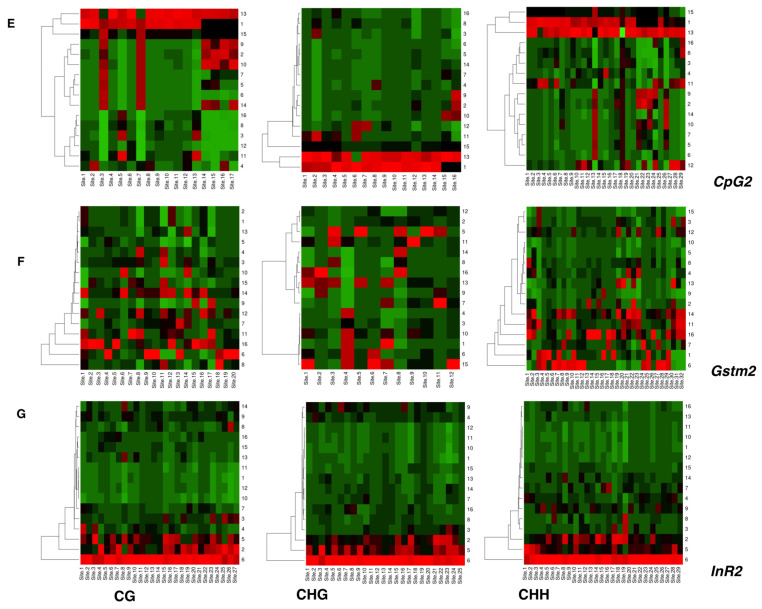
Site-specific analysis of the heritability of stress-induced methylation marks in BPH. Heatmaps were plotted for the cytosine sites (within CpG islands corresponding to stress-responsive genes; **A**–**G**) that exhibited significant variation between samples, in all three contexts. Gene names are mentioned at the right, and context information is provided at the bottom. Numbers on the right of each plot correspond to sample names, which are as follows: 1. BOD_AD 2. BOD_NY 3. PR 4. RG1 5. RG4 6. RNG1 7. RNG4 8. PI 9. IG1 10. IG4 11. ING1 12. ING4 13. RT1 14. RT2 15. IR1 16. IR2. The colour scale represents an increase in methylation from red to green.

Heatmaps plotted for cytosine sites that exhibited significant variation between samples confirmed that these stress-induced epigenetic marks (in both CG and non-CG contexts) in BPH are heritable across generations (Figure 8). It was observed that this pattern is inconsistent across genes and differs based on the nature of the stresses. After removal of stress (imidacloprid), *CYP6AY1* demonstrated intergenerational transfer of some of the stress-induced methylation marks to the progeny (F1); however, these marks faded in the subsequent generation (i.e., F2 progeny) (Figure 8A). No inheritance (i.e., transfer of methylation marks) was observed for *CYP6AY1* in RH-exposed BPH samples. In contrast, *EG* showed complete transfer of all the stress-induced epigenetic marks to the future generations, i.e., F1 and F2 progeny, for pesticide and nutritional stress (Figure 8B).

In the case of *Est*, BPH adults showed uniform hypomethylation of cytosines across all sites, irrespective of the treatment (i.e., for control, RH, and pesticide-exposed insects), but nymphs showed hypomethylation of this locus only under stress (Figure 8C). However, removal/withdrawal of stress (RH) increased the methylation status of this locus, comparable to that of nymphs under control conditions, both in F1 and F2 generations. In addition, as observed in the case of *CYP6AY1*, *Est* also demonstrated partial reversals in the F1 and F2 generations (under imidacloprid stress; Figure 8C). 

*CYP6ER1*-*CpG1* displayed inconsistent and heterogeneous methylation across sites, especially in CG and CHG contexts, some of which were heritable across generations (Figure 8D). However, the cytosines were mostly hypermethylated in the CHH context, except for some sites located at the 5′ end of the CpG island, which showed heritability (Figure 8D). Similarly, *CYP6ER1-CpG2* also exhibited heterogeneous methylation patterns across sites and treatments in all three contexts (Figure 8E). Further, and in accordance with our previous analyses based on overall percent methylation, it was observed that *InR2* and *Gstm2* (Figure 4 and Figure 5) largely exist in the hypermethylated state across treatments, in all three contexts (Figure 8F,G). However, the site-specific analysis revealed a slight dip in the methylation status of *InR2* (in ~3–5% of the total reads) for BOD nymphs, across some sites, in all three contexts. Furthermore, under RH stress, nymphs (1st generation, RNG1) displayed complete hypomethylation of this locus across all sites (Figure 8G). 

### 2.5. Azacytidine Assay

**Figure 9:**
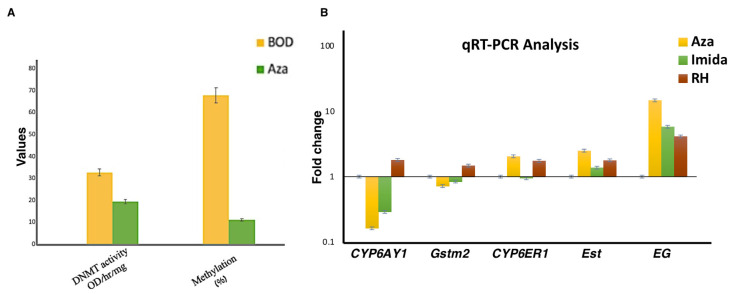
Effect of pharmacological disruption of methylome (5′-azacytidine treatment) on BPH (**A**) Bar plots showing reduction in cellular DNMT levels and percent methylation across stress-responsive genes in BPH after treatment with azacytidine. Error bars represent SD. (**B**) Quantitative RT-PCR-based analysis of gene expression performed for BPH populations after treatment with azacytidine and upon exposure to stress (Imidacloprid and nutritional stress). BOD population (untreated and unexposed to stress) was used as a control. The relative gene expression was normalised to the expression level of *Actin*. The results were analysed using the 2^−ΔΔCt^ method, and the expression level was displayed as relative expression values based on the relative standard curve method. The analysis was based on three biological and three technical replicates.

DNMT activity assay indicated ca. 40% reduction in DNMT activity following 5-aza treatment (Figure 9A). Further, quantification of methylation levels using a methylation-sensitive restriction digestion assay revealed a significant drop in methylation levels of BPH genes (the levels dipped from 70% to 12%; Table S6) after azacytidine treatment, further confirming the efficiency and reliability of this treatment (Figure 9A). 

### 2.6. Effect of Methylation on Gene Expression as Revealed by qRT-PCR Analysis

Gene expression analysis revealed significant changes in the mRNA levels in BPH after treatment with 5-aza and upon exposure to stress (Figure 9B). In 5-aza-treated insects, while the expression levels of *CYP6ER1* and *Est* increased by >2-fold, *EG* showed a more than 14-fold increase, thereby implying that methylation negatively regulates the expression of these genes. This was further supported by the fact that *EG* exhibited a hypomethylated state under stress (similar to what was observed after azacytidine treatment) and showed >4-fold and a 5-fold increase in its expression after exposure to RH and imidacloprid, respectively (Figure 9B). However, and in contrast, *CYP6AY1* showed significant downregulation in its expression (i.e., >6-fold decrease) after treatment with azacytidine and also upon exposure to stress (imidacloprid; 3.46-fold reduction). Further, *Gstm2* did not show any significant change in its mRNA levels upon exposure to stress (Imidacloprid and RH) and/or after treatment with 5-aza (Figure 9B). Furthermore, correlation analysis of methylation values and expression data indicated that, while expression levels of *CYP6ER1*, *EG*, and *Est* exhibited a strong negative correlation with methylation (correlation coefficient −0.635, −0.883, and −0.895 for *CYP6ER1*, *EG*, and *Est*, respectively), *Gstm2* and *CYP6AY1* showed a weak positive correlation (correlation coefficient 0.548 and 0.270 for *Gstm2* and *CYP6AY1,* respectively).

## 3. Discussion

Epigenetic mechanisms act at the genotype-to-phenotype interface, where they regulate various developmental and physiological processes, mediate responses to external environments, and sometimes even determine the inheritance of gene expression patterns [39]. Although, over the past few decades, these mechanisms have been extensively studied in higher organisms (including humans and plants), we still have limited information on their existence in, and relevance for, insects. It is widely observed that insects have the capacity to rapidly adapt to changing environmental conditions and colonise diverse ecological niches. While it is known that genome plasticity underpins their rapid adaptive nature, we are yet to fully understand the molecular mechanism(s) involved in transducing the environmental stimuli into induced genetic and phenotypic changes, enabling their survival under extreme environments. Although the physiological and biochemical processes in an organism are primarily determined by its inherited genetic makeup, the genome structure and function could also be controlled by epigenetic processes, such as DNA methylation. Hence, we hypothesised that changes in the status of DNA methylation could lead to the formation of new and heritable phenotypes while widening the source of genetic and phenotypic variations within insect populations and, consequently, favouring adaptation. With this view and using BPH as the model organism, the current study focused on the role of DNA methylation in conferring genetic plasticity to insect populations (including their ability to rapidly build up resistance against any pesticide and/or rapidly overcome host plant resistance). 

### 3.1. Global Methylation Analysis Revealed Epigenetic Reprogramming of the BPH Genome in Response to Pesticide and Nutritional Stresses

Quantification of 5-methylcytosine in BPH adults and nymphs revealed differences in DNA methylation levels across life stages. It was observed that nymphs had a higher amount of DNA methylation as compared to adults. Here, it is worth noting that DNA methylation mediates phenotypic polymorphism in insects by regulating complex cellular processes, such as cell differentiation and development [40]. For instance, it has been shown that DNA methylation levels are linked to the development of winged and wingless forms in aphids [41], brain development, memory function and behaviour in honey bees [42], and caste formation in ants [38]. All these phenomena (i.e., cellular differentiation, brain development, and caste formation) occur during the early developmental stages. Therefore, and in concurrence with observations made for other insect species [7,43], it is possible that methylation is involved in morph differentiation (macropterous and brachypterous), growth and development in BPH nymphs, which, in turn, explains its differential methylation across the life-stages of BPH. However, to substantiate this, it is crucial to study the methylation status of genes involved in morph development and differentiation in BPH across its life stages. 

Next, we observed the differential response of nymphs and adults, vis-à-vis their methylation status, to both pesticide and nutritional stresses. Compared to adults, nymphs displayed a slightly delayed response to stress (i.e., alteration of methylation status at the nymphal stage was observed only after prolonged exposure of insects to stress, i.e., by 4th generation; Figure 2). This indicated that while BPH at the adult stage can immediately sense and respond to various stresses, at the nymphal stage, it probably prefers to invest its energy and resources in other crucial growth and developmental processes till such time that its survival is at stake. Therefore, it is only after prolonged and continued exposure to stress conditions that BPH individuals display uniform alterations in methylation status across different life stages. However, due to insufficient experimental evidence, this is merely speculation. 

Further, results obtained from the global DNA methylation analysis indicated that BPH responds differentially to pesticide and nutritional stress. While exposure to imidacloprid resulted in an overall increase in percent methylation, nutritional stress (RH) caused hypomethylation of the BPH genome, suggesting that the epigenetic reprogramming in BPH is stress-specific. Furthermore, we observed that BPH populations maintained their altered methylation status while the stressors (RH and imidacloprid) persisted. Moreover, upon returning to favourable conditions, insects did not show an immediate but a gradual reversal of their methylation status over a span of two generations, thereby hinting at the persistence of stress-induced epigenetic changes in BPH (Figure 2). To corroborate these possibilities and elucidate the role of methylation in the observed stress-resistance of BPH, a set of stress-responsive genes was screened for stress-induced methylation patterns and their heritability across generations. 

### 3.2. Demethylation/Methylation of Stress-Responsive Genes Is Stress- and Life Stage-Specific and Is Likely Determined by Their Functional Relevance for BPH Survival under Hostile Environments

Alteration in the expression levels of *CYP6AY1*, *CYP6ER1*, *Carboxylesterase*, *Gstm2, InR2,* and *Endoglucanase* (i.e., genes shortlisted for investigation in the current study) in response to pesticide and nutritional stress is well documented [44,45,46,47,48,49]. However, the molecular mechanism(s) regulating their activity in response to external stimuli remain largely unexplored. Here, we hypothesised the involvement of DNA methylation in modulating their activity in response to environmental cues. In this regard, we found that the CpG islands (regions prone to methylation) within these genes are mainly localised to the gene body and contain various regulatory motifs and splice sites (Figure 3), which further implied that demethylation/methylation of these islands could significantly impact/regulate gene activity and function. In addition, several mRNA variants of *CYP6ER1* have been reported [50]. Therefore, the possibility that this situation arose as a consequence of differential methylation of this gene under different physiological conditions is high. However, to validate and confirm these speculations, we employed a targeted bisulfite sequencing technique to study alterations in the methylation patterns of stress-responsive genes upon exposure to stress. 

Our data revealed that the methylation status of BPH genes is indeed influenced by external environmental conditions. We found distinct patterns of methylation in CG and non-CG contexts under pesticide and nutritional stresses (Figure 6). This was in congruence with our previous study, where the prevalence of methylation in all three contexts (i.e., CG, CHG, and CHH) in the BPH genome was reported [51]. It was observed that upon exposure to stress (imidacloprid and RH), the methylation state of *CYP6AY1* and *CYP6ER1*-*CpG1* changed remarkably in all three contexts, while *Rep1* showed variation only in the CHH context (Figure 4). Being an intragenic tandem repeat element, the methylation/demethylation of *Rep1* is likely associated with a different method of gene regulation, which also explains its unique methylation pattern (i.e., changes restricted to CHH context) in response to stress. In humans, it has already been established that the intragenic tandem repeats operate as expression and methylation quantitative trait loci, and, therefore, variations in their methylation level significantly impact gene expression and genome architecture [52]. It is likely that a similar phenomenon exists in insects, but this warrants further investigation. Unlike *Rep1*, methylation levels of *Rep2* did not show any stress-induced alterations but displayed variations across life stages (i.e., between adults and nymphs), suggesting their likely involvement in BPH growth and development as well (Figure 4). However, to fully understand and ascertain the role of methylation/demethylation of intragenic repeat elements in insects, it is crucial to analyse other repeat elements present in the BPH genome and extend such studies to other insect species.

Further, *Gstm2* and *InR2* did not display any significant change in their methylation status under both pesticide and nutritional stress, thus suggesting that either these genes do not respond to these (pesticide and nutritional) stresses or their activity is not regulated by methylation. However, and in contrast, the methylation pattern of *Est* (in adults) and *CYP6ER1*-*CpG2* (both in adults and nymphs) was altered only in response to nutritional stress, while *EG* changed only under pesticide stress (Figure 4). Collectively, this suggests that the methylation status of BPH genes is probably determined by its functional relevance for BPH survival under any given physiological condition. Such indications also came from our observation that *CYP6AY1* and *CYP6ER1* displayed significant methylation changes upon exposure to pesticide and nutritional stresses. Here, it is worth noting that *CYP6AY1* and *CYP6ER1* belong to the family of P450 monooxygenases and are involved in the detoxification of several xenobiotics (particularly imidacloprid) and virulence adaptation in BPH [50,53]. Therefore, it is possible that exposure to both imidacloprid and resistant rice variety (RH) altered their methylation state. Besides, duplication and neofunctionalisation events have been reported for *CYP6ER1* [50]. In our study, *CYP6ER1*-*CpG1* represented a region unique to the mRNA variant (*CYP6ER1vA*) primarily involved in imidacloprid detoxification [50], but *CYP6ER1*-*CpG2* shared homology with *CYP6ER1vL* and *CYP6ER1vF*. As *CYP6ER1-CpG2* displayed variation under nutritional stress, *CYP6ER1vL* and *CYP6ER1vF* are likely involved in the detoxification of host metabolites, but this needs to be experimentally demonstrated. Nevertheless, this strengthens our assumption that the methylation status of BPH genes is determined by their functional significance to BPH under any particular environmental condition. Furthermore, the estimation of variability in methylation scores for each gene under pesticide and nutritional stress using whisker plot analysis also supported the suggestion that the methylation status of genes in BPH is stress-specific (Figure 5C,D).

In addition, the delineation of the influence of two experimental variables (i.e., life stages and stresses) on the methylation status of BPH genes revealed that although adults and nymphs differ in their methylation status under normal (favourable) conditions, they exhibit a similar ‘epigenotype’ under stressed conditions (Figure 7). This is in agreement with our earlier findings, based on global DNA methylation analysis, where we observed that BPH displays uniform alterations in methylation status across its different life stages after exposure to stress conditions. However, it is interesting to note that while the changes in methylation at a global scale were evident only after 4 generations of continuous exposure, stress-responsive genes, in contrast, demonstrated a very rapid response, i.e., alteration in their methylation status, within 10 days of exposure (Figure 7). Considering that the initiation of epigenetic reprogramming is an energy-expensive phenomenon, it is plausible to expect changes localised to certain regions of the BPH genome (primarily limited to the genes directly involved in stress resistance) than on the global scale, especially during the initial periods of exposure. 

Further, it was observed that nymphs (1st generation) feeding on RH mounted a stronger initial response than adults, which declined gradually by the 4th generation (Figure 7). This was probably because nymphs allocate much of their resources towards feeding to ensure their survival to the adult (reproductive) stage. Hence, they exhibited a stronger response *viz.* epigenetic changes, which likely increased their survival on, and capacity to tolerate resistant host plants. Earlier studies have indeed demonstrated this, where it has been experimentally shown that nymphs usually outperform adults on resistant rice varieties [54]. 

Additionally, our data also indicated that nutritional stress was a relatively stronger stimulus for inducing epigenetic changes in BPH compared to pesticide stress. Insects under pesticide stress had higher correlation values than those feeding on RH (Figure 7). This is perhaps due to the fact that, apart from metabolic detoxification, insects have evolved multiple ways to resist/tolerate pesticides, such as penetration resistance, target-site alteration, and behavioural resistance [55,56]. This is in contrast to the breakdown of host plant resistance that is singularly dependent upon the metabolic processes of the insect [57]. Therefore, it is possible that feeding on resistant rice variety (RH) induces a faster and more robust epigenetic response as compared to pesticide exposure, which needs to be investigated further. Nevertheless, based on these results, it is plausible to state that methylation dynamics in BPH is governed by the nature of stressor(s) and is life-stage dependent. 

A unique feature of these environmentally-induced modifications is their propensity for getting transmitted to the next generation and, in the process, enabling them to be pre-endowed with the capacity to resist/overcome stress and/or mount a stronger and faster response should they encounter similar stresses during their life cycle. In this regard, our analyses, both at the global and gene-specific levels, hinted at the heritability of pesticide-induced changes in DNA methylation across generations (Figure 4A and Figure 6E). However, these changes underwent immediate reversal in the case of nutritional stress (Figure 4B and Figure 6F) when the stressor was removed. Therefore, to ascertain the consistency of this phenomenon across genes and stresses and check whether these stress-induced epigenetic marks get uniformly passed on to subsequent generations, we studied the heritability of these methylation marks at the nucleotide level.

### 3.3. Stress-Induced Methylation Marks in BPH Are Heritable

Our study revealed the heritability of stress-induced epigenetic marks in BPH (in both CG and non-CG contexts) across generations (Figure 8). However, this pattern was inconsistent across genes and differed based on the nature of the stresses. Although the stress-induced (imidacloprid) methylation marks (as in the case of *CYP6AY1*) persisted in the F1 progeny, even in the absence of stress, they faded in the subsequent generation (i.e., F2 progeny) (Figure 8A). No inheritance (i.e., transfer of methylation marks) was observed for *CYP6AY1* in the case of nutritional stress. In contrast, *EG* showed complete transfer of all the stress-induced epigenetic marks to future generations, i.e., F1 and F2 progeny, for pesticide and nutritional stress (Figure 8B). While this hinted at the involvement of methylation in defence priming, the fact that these marks ‘faded’ in the absence of stress (as in the case of *CYP6AY1*) also indicated the existence of a trade-off and/or fitness cost associated with the maintenance of a primed epigenetic state. However, to confirm this, it is important to first study life-history trade-offs and fitness cost(s) associated with epigenetic reprogramming in BPH.

Further, the study of methylation patterns of *Est* revealed that epigenetic response(s) in BPH also depends on its life stage. We observed that while BPH adults exhibited uniform hypomethylation of cytosines across all sites and treatments (i.e., for control, RH, and pesticide-exposed insects), nymphs displayed hypomethylation only under stressed environments (Figure 8C). Further, removal/withdrawal of nutritional stress (RH) increased the methylation status of this locus, comparable to that of nymphs under control conditions, both in the F1 and F2 generations. Although at this juncture, it is difficult to discern the likely implications of these observations, due to the limited information available, it emphasises the importance of considering juvenile life stages while studying adaptive stress responses via epigenomic changes in BPH. In addition, as observed in the case of *CYP6AY1, Est* also demonstrated partial reversals in the F1 and F2 generations (under imidacloprid stress), further supporting the idea of epigenetic inheritance in BPH (Figure 8C). 

*CYP6ER1*-*CpG1* displayed inconsistent and heterogeneous methylation across sites, especially in CG and CHG contexts, and some of these were heritable across generations (Figure 8D). However, in the CHH context, the cytosines were mostly hypermethylated, except for some sites located at the 5′ end of the CpG island, which showed heritability (Figure 8D). Interestingly, it has been suggested that stress-induced DNA methylation is largely concentrated in the non-CG context [58]. Non-CG methylation is known to play a major role in transposon and gene silencing, maintaining genome integrity, and mediating environmental responses [59]. Besides, while CG methylation (being symmetrical) can be successfully transmitted during replication, asymmetric methylation (i.e., methylation in non-CG contexts) is maintained by the persistent activity of de novo methyltransferases [60]. This further adds to the possibility of their larger contribution towards mediating stress responses. However, we still have much to learn regarding its relevance and role in BPH. 

Further, it was observed that *CYP6ER1-CpG1* and *CYP6ER1-CpG2* also exhibited heterogeneous methylation patterns across sites and between treatments in all three contexts (Figure 8E). Heterogeneous methylation across sites could be attributed to the BPH genome containing several copies of *CYP6ER1*, each having a different base composition and representing a unique variant of this gene involved in diverse metabolic pathways [50]. Therefore, while exposure to pesticide and nutritional stress likely caused variation in the methylation status in some variants, others likely remained unaltered. Since our analysis design is targeted at the CpG islands within *CYP6ER1*, which are mostly conserved across all *CYP6ER1* variants, it is therefore likely that our data is derived across different variants of this gene and, as a result, could account for some of the heterogeneity observed in its methylation status.

Additionally, and in agreement with our data on the overall percent methylation, it was observed that *InR2* and *Gstm2* (Figure 4 and Figure 5) largely existed in the hypermethylated state across treatments, in all three contexts (Figure 8F,G). However, the site-specific analysis revealed a slight dip in the methylation status of *InR2* for BOD nymphs, across some sites, in all three contexts. Under RH stress, nymphs (1st generation, RNG1) displayed complete hypomethylation of this locus across all sites (Figure 8G). However, this change (hypomethylation) was restricted to a few reads (i.e., ~3–5% of the total reads obtained for RNG1). Interestingly, *InR2* is predominately expressed in the wing buds at the nymphal stage and regulates wing polyphenisms [45] and morph switch in BPH [61]. It has been shown that wing morph determination in BPH is controlled by a nutrient-sensing pathway. Given the tissue-specific activity (expression) of *InR2* and the fact that morph differentiation at the nymphal stage is determined by host quality, the observed changes in its epigenetic status in nymphs under nutritional stress were expected.

Taken together, the site-specific analysis not only confirmed life-stage-specific differences in the methylation pattern across BPH genes in response to changing environmental conditions but also revealed their inheritance across generations. Further, as these changes took place within a very short time frame, we hypothesised that they confer rapid adaptive capacity and stress resilience to BPH. However, to corroborate such a possibility, it is important to assess if alteration(s) in methylation status could impact the expression (activity) of the genes screened in this study, which we subsequently demonstrated. 

### 3.4. Azacytidine Treatment Caused a Reduction in Cellular DNMT Levels Leading to Hypomethylation of BPH Genes

Azacytidine (5-azacytidine; 5-aza), an inhibitor of DNA methyltransferase, at concentrations >40 μM, was lethal for BPH, suggesting that methylation is vital for BPH and disruption of its methylome beyond a certain threshold impacts its survival. Further, to investigate whether the activity of the selected stress-responsive genes is modulated by methylation, BPH individuals were treated with azacytidine (40 μM), and the respective transcript profiles of the selected genes were determined. However, prior to assessing gene expression profiles, we ensured that exposure to azacytidine indeed impacted the methylation status of genes under consideration in the present study. This was important because azacytidine (a cytidine analogue) acts only when incorporated into DNA during replication, repair, transcription, or demethylation (i.e., processes involving nucleotide replacement). When DNMTs encounter these cytidine analogues, they get irreversibly and covalently bound to the inhibitor, consequently depleting the cell of its DNMT pool. This implies that azacytidine can only inhibit methylation in regions where they are incorporated and where DNMT can access them. Therefore, it was crucial to evaluate cellular DNMT activity and the methylation status of these stress-responsive genes, following treatment with 5-aza, before assessing its impact on transcription. In this regard, a DNMT activity assay indicated ca. 40% reduction in DNMT activity following 5-aza treatment (Figure 9A). Further, quantification of methylation levels using a methylation-sensitive restriction digestion assay revealed a significant drop in methylation levels of BPH genes (the levels dipped from 70% to 12%; S6) after azacytidine treatment, further confirming the efficiency and reliability of this treatment (Figure 9A). 

### 3.5. Methylation Influences Gene Expression in BPH 

The pharmacological erasure of the BPH methylome with azacytidine led to transcriptional changes in BPH. Upon treatment with 5-aza, the expression levels of *CYP6ER1* and *Est* increased by >2-fold, whereas *EG* showed a more than 14-fold increase, implying that methylation negatively regulates the expression of these genes. This was further corroborated by the fact that *EG* exhibited a hypomethylated state under stress (similar to what was observed after azacytidine treatment) and showed >4- and 5-fold increase in its expression after exposure to RH and imidacloprid, respectively (Figure 9B). However, in contrast, the reduction of 5 mC after the treatment with the methylation inhibitor led to reduced *CYP6AY1* transcripts. A plausible explanation for this observation could be that the activity of this gene is regulated by other molecular mechanisms that are epigenetically controlled. Further, it has already been observed in many organisms that the influence of gene regulation through DNA methylation also depends on the context of the surrounding genes [62,63] and other epigenetic processes, such as histone acetylation and chromatin architecture [64]. Therefore, it is possible that similar mechanisms also operate in BPH and likely control the expression of *CYP6AY1*, which warrants further investigation. 

Next, the inhibition of DNA methylation (due to azacytidine treatment) or exposure to stress (both pesticide and RH) did not affect the transcription of *Gstm2.* In congruence with these observations, we also did not observe any modification(s) in the methylation status of *Gstm2* upon exposure to stress (pesticide and RH), further indicating that the activity (expression) of this gene is likely not regulated by methylation. While it is well established that DNA methylation is one of the several molecular mechanisms that cells use to regulate gene expression, our results indicated that the roles and targets of DNA methylation differ depending on the genes under study. We observed that methylation likely influences the expression of some of the genes analysed in this study. To the best of our knowledge, this is the first report that indicates how crucial genes that likely contribute to the rapid adaptive response in BPH are influenced by its methylation status.

Furthermore, correlation analysis of methylation values and expression data indicated that while expression levels of *CYP6ER1*, *EG,* and *Est* exhibited a strong negative correlation with methylation (correlation coefficient −0.635, −0.883, and −0.895 for *CYP6ER1*, *EG,* and *Est*, respectively), *Gstm2* and *CYP6AY1* showed a weak positive correlation (correlation coefficient 0.548 and 0.270 for *Gstm2* and *CYP6AY1,* respectively). It is widely known that DNA methylation influences gene expression by affecting interactions of DNA with transcription factors, regulatory elements, and chromatin proteins [65]. Therefore, it is plausible to consider methylation as a negative regulator of gene expression. However, considering the lack of adequate information, the complexity of the epigenetic systems, and the fact that different epigenetic mechanisms are highly intertwined, we believe it is important to obtain a deeper understanding of this process to establish the relationship between DNA methylation and transcription. 

Collectively, this investigation into the effect of pesticide exposure and host plant resistance on the BPH methylome revealed that the pattern(s) of changes in DNA methylation in response to environmental cues likely regulates/regulate its defence response and resistance to various stresses and is/are life-stage dependent. However, in the current study, we recorded changes in the methylation pattern in BPH in response to two stresses (pesticide and nutritional). While exposure to imidacloprid resulted in an overall increase in percent methylation, nutritional stress (RH) caused hypomethylation of the BPH genome. Therefore, it would be interesting to observe whether the same methylation pattern(s) would ensue when BPH individuals are exposed to two or more stresses simultaneously, especially where the influence of the stresses is contrasting with regard to their effect on the BPH epigenome. 

Further, our findings provide novel insights into the heritability of stress-induced methylation marks in BPH. We showed that environmentally triggered epigenomic changes in BPH are selectively transmitted across generations. However, it was also observed that stress-induced perturbations in DNA methylation in BPH, though heritable, eventually fade in the absence of the stressor, thereby hinting at the likely existence of fitness cost(s) associated with the maintenance of a stressed epigenotype. This is in congruence with a widely accepted notion that epigenetic modifications sometimes bestow fitness costs upon the offspring, which might last for several generations. Hence, BPH reverted to its normal methylation profile in the absence of any selection pressure (i.e., stress). In this regard, a deeper investigation into assessing life-history trade-offs and fitness costs underlying epigenetic inheritance in BPH would provide us with a deeper and definite basis for understanding this phenomenon. As the current study includes comparative analyses of the methylation patterns restricted to a set of selected stress-responsive genes, future studies involving epigenome-wide changes in DNA methylation patterns in response to environmental cues are warranted. 

Furthermore, we demonstrated how DNA methylation influences the expression levels of some stress-responsive genes in BPH. To the best of our knowledge, this study, for the first time, highlighted demethylation/methylation as a phenomenon underlying rapid adaptive phenotypes and observed genetic plasticity in BPH populations, thereby adding to our current understanding of the molecular processes that enhance the ability of BPH to rapidly cope with, and adapt to, a dynamic environment. In summary, as a proof-of-concept study, elucidating the role of epigenomic modifications in transducing environmental stimuli into heritable changes that can likely alleviate stress and favour insect survival, offers a precise and targeted approach to tackling pest outbreaks. The insights gained from this study not only have implications for the management of this destructive pest in a sustainable and environmentally-friendly manner, but the findings thus obtained could also serve as the basis for extending such investigations to other important pests. 

## 4. Materials and Methods 

### 4.1. BPH Populations

A BPH population was raised on TN1 (Taichung Native 1; a susceptible rice variety) in a BOD incubator maintained at 28 °C with 16 h light and 8 h dark photoperiod at ICGEB, New Delhi, for over 30 generations. This highly inbred population was initiated from 2 males and 2 females collected in 2017 from the rice fields in Delhi, and it served both as the control (unexposed to stress) and the source population for obtaining stress-exposed insects. BPH individuals (>50) from the control population (BOD) were randomly collected for stress exposure (pesticide and nutritional stress). 

### 4.2. Pesticide Stress

Prior to pesticide exposure, LD_50_/LC_50_ imidacloprid (Confidor 17.80% SL; Bayer AG, Leverkusen, Germany) was estimated for the BOD population to ascertain its pesticide resistance/tolerance status. Additionally, field-collected BPH insects were also subjected to LD_50_ analysis and used as a comparator to confirm if the laboratory-generated (BOD) population was susceptible to imidacloprid. For LD_50_ analysis, BOD insects (adults) were anesthetised on ice for 3–4 min, followed by topical application of the pesticide solution (0.5 µL/insect) comprising different concentrations of imidacloprid (active ingredient (a.i.) 0.5–4.0 ng). Post-treatment, these insects were released onto TN1 plants in a cage, and the number of live insects was counted 24 and 48 h after treatment. For LC_50_ estimation, control insects (BOD population) were released onto TN1 plants sprayed with different concentrations of pesticide (ranging from 0.02–0.5% imidacloprid). Live insects were counted 24 and 48 h after release. The LD_50_/LC_50_ data were subjected to probit analysis using SPSS Statistics (v. 22.0; IBM, Armonk, NY, USA). Both LD_50_/LC_50_ experiments were performed in triplicates with 10 insects per replicate and for each concentration. 

Thereafter, we generated a pesticide-resistant BPH population in the laboratory by periodically exposing a set of individuals from the BOD population (~50; adults) to LC_40_ imidacloprid solution (0.087% Confidor) for the first two generations, which was subsequently increased to LC_60_ imidacloprid (0.162% Confidor). Insects would be sprayed with the pesticide solution twice a week using a spray bottle. From this population, individuals representing different stages in the BPH life-cycle were randomly collected and used for this study. BPH insects collected 7 days after pesticide exposure were regarded as the parental population (PI). The subsequent 1st and 4th generation BPH adults arising from PI were termed IG1 and IG4, respectively. In addition, 2nd/3rd instar nymphs from the 1st and 4th generations were also collected and referred to as ING1 and ING4, respectively. Subsequently, insects (4th generation; adults) under pesticide stress were transferred back to the normal conditions (i.e., onto TN1 plants and with no further exposure to pesticide). Collections were made for 1st and 2nd generation adults (arising from individuals brought back to control conditions) and were referred to as IR1 and IR2, respectively. All collected insects were preserved in absolute ethanol (99.9%) and stored at −20 °C till further use. 

### 4.3. Nutritional Stress

BOD individuals (~50; adults) were exposed to nutritional stress by transferring them onto RH (Rathu Heenati; a resistant rice variety) plants. BPH (8–10 individuals) feeding on RH plants were collected at different time points and across different life stages. Insects collected 7 days after feeding on RH were referred to as the parental population (PR), while subsequent 1st and 4th generation BPH adults, arising from PR, were termed RG1 and RG4, respectively. In addition, 2nd/3rd instar nymphs from the 1st and 4th generations were collected and referred to as RNG1 and RNG4, respectively. Subsequently, insects (4th generation; adults) feeding on RH were transferred back onto the susceptible rice variety TN1. Collections were also made representing 1st and 2nd generation adults (arising from individuals brought back to TN1) and referred to as RT1 and RT2, respectively. All collected insects were preserved, as stated above.

### 4.4. Screening for Callose Deposition

In rice, blockage of sieve tube elements due to callose deposition is a common resistance response triggered/induced by BPH feeding [66]. Hence, the formation of callose plugs was taken as an indication of feeding and used as a marker to ensure that BPH populations analysed in the present study had fed on RH plants. Callose deposition in the RH plants (exposed to BPH population; PR) was observed and compared with the TN1 plants using the aniline blue staining method [67], with slight modifications. Here, plant tissue samples (representing the region near the base of the stem; feeding site of BPH) of 15-day-old RH and TN1 seedlings (10 days after exposure to BPH) were decolorised using an acetic acid and ethanol mix (3:1), equilibrated with 0.15 M phosphate buffer, followed by hand sectioning. The stem sections (from RH and TN1 plants) were then stained with 150 mM aniline blue solution for 30 min, mounted on glass slides, and examined under the UV fluorescent light (DAPI filter; excitation filter 390 nm, dichroic mirror 420 nm, and emission filter 460 nm) using a confocal microscope (A1R, Nikon, Tokyo, Japan). The amount of callose deposition was evaluated by counting the number of sieve plates that showed thick callose deposition. Photographs were taken in brightfield and fluorescence modes. 

### 4.5. Isolation of BPH Genomic DNA

Total genomic DNA was extracted from eight individual insects of each population (for details, see Table S3) using the GF-1 tissue DNA extraction kit (Vivantis, Selangor, Malaysia), following the manufacturer’s instructions. The isolated DNA was quantified using the NanoDrop Spectrophotometer (Thermo Fisher Scientific, Waltham, MA, USA), and the quality was checked by electrophoresis on 0.8% TBE agarose gel [68]. 

### 4.6. Global DNA Methylation Analysis

Quantitating DNA methylation is a prerequisite for understanding and targeting DNA methylation-associated changes in an organism. With this view in mind, global DNA methylation analysis was carried out for BPH populations (for details, see ‘Table S3′) using MethylFlash Global DNA Methylation (5-mC) ELISA Easy Kit (Colorimetric) (Epigentek, Farmingdale, NY, USA), following the manufacturer’s protocol. The change in the amount of 5-methylcytosine in the BPH genome upon exposure to stress was estimated by comparing with respective controls. 

### 4.7. Screening of Stress-Associated BPH Genes for Their Methylation Status

A set of stress-responsive genes in BPH, known to confer resistance/tolerance to stress, were screened for stress-induced methylation marks using the targeted bisulfite sequencing technique. However, as previous studies have revealed very low levels of methylation in insects, predominantly confined to the CpG islands [10], we restricted our analysis of the methylation status to the CpG islands within the shortlisted-stress-responsive genes (Table S1). The procedure involved bisulfite modification of DNA prior to PCR amplification and sequencing (details below) and is based on the premise that while the treatment would convert all unmethylated cytosines to uracil, which are read as thymine during PCR amplification, the methylated (5-methylcytosines) cytosines would remain unaltered. Therefore, upon sequencing, the presence of cytosine at any given site would reflect its methylated state. Hence, methylation marks were detected using this technique at single base-pair resolution. 

#### 4.7.1. Prediction and Characterisation of CpG Islands and Primer Design

Selected genes were screened for the presence of CpG islands (i.e., regions prone to methylation) using EMBOSS CpGplot (https://www.ebi.ac.uk/Tools/seqstats/emboss_cpgplot/; Accessed on 15 July 2018). Identification parameters included the ratio of observed CpG to expected CpG (Obs/Exp value >0.6), sequence length (>200 bp), and the GC content (>50%). Further, as it is already well established that methylation of CpG islands can alter the binding of transcription factors, influence gene expression, and also result in the generation of splice variants, we screened the identified CpG regions in each of the selected genes for the presence of regulatory motifs (such as CTCF motifs), transcription factor binding sites (TFBS) and alternate splice sites using CTCFBSDB 2.0 (https://insulatordb.uthsc.edu/; Accessed on 21 March 2020), Nsite tool hosted by Softberry (http://www.softberry.com/berry.phtml?topic=nsite&group=programs&subgroup=promoter; Accessed on 21 March 2020), and BDGP (https://www.fruitfly.org/seq_tools/splice.html; Accessed on 21 March 2020), respectively. 

Next, the bisulfite-PCR primers for each gene were designed from regions flanking their respective CpG islands. Considering that after bisulfite conversion, DNA strands lose complementarity to each other, primers were designed such that they could amplify both the converted and non-converted template in an unbiased manner. The primers were designed using MethPrimer (https://www.urogene.org/methprimer/; Accessed on 19 January 2021) and the MacVector suite of sequence analysis programmes (MacVector Inc., Cary, NC, USA; version 15.5). The sequence information of all the primer pairs used in this study for methylation analysis is provided in Table S7. 

#### 4.7.2. Bisulfite Conversion of BPH DNA

Genomic DNA isolated from eight individuals of each population was pooled in equal amounts for the bisulfite treatment. Bisulfite conversion was performed using 200 ng pooled DNA/reaction using EpiJET Bisulfite Conversion Kit (Thermo Scientific, Waltham, MA, USA) as per the manufacturer’s protocol. DNA recovery after bisulfite treatment was assessed using a Qubit 4.0 fluorometer (using dsDNA assay and ssDNA assay kits; Thermo Fisher Scientific, Waltham, MA, USA), and conversion efficiency was estimated based on the conversion rate of the control reaction. The control reaction consisted of a PCR product (283 bp, corresponding to a portion of the Actin gene of BPH) generated using ACT-mod F 5′ TGCGTGACATCAAGGAGAAGCTG 3′ and ACT-mod R 5′ GTACCACCGGACAGGACAGT 3’ primers. The PCR conditions were as follows: initial denaturation for 2 min at 94 °C, followed by 30 cycles of 30 s at 94 °C, 30 s at 55 °C, 30 s at 72 °C, and final elongation at 72 °C for 2 min. The PCR reaction (25 μL) consisted of a 25 ng DNA template, 200 μM dNTPs, 0.6 U Taq DNA polymerase, 1X Taq buffer, and 13 μM each of forward and reverse primers. The PCR product was separated on 1% agarose gel and purified using a nucleic acid extraction kit (Vivantis GF-1, Subang Jaya, Malaysia). As the PCR amplified fragment is essentially devoid of methylation, all the cytosines in the fragment were expected to be converted to uracil during the bisulfite treatment. Therefore, this fragment was processed simultaneously with the test samples and served as the control for bisulfite treatments. The control DNA was again PCR amplified using the same primer pair post bisulfite treatment. The PCR product thus obtained was cloned into a pCR4–TOPO vector using TOPO TA Cloning Kit (Invitrogen, Waltham, MA, USA). Plasmid DNA was isolated from positive clones and sent to M/s Macrogen Inc. (Seoul, Korea) for Sanger sequencing. The chromatograms were evaluated using the MacVector suite of Sequence Analysis Programmes (MacVector Inc., Cary, NC, USA; version 15.5). Based on the comparison of the sequencing data of the treated with that of the untreated control fragment, the bisulfite conversion efficiency was calculated as a ratio of converted to non-converted cytosines. 

#### 4.7.3. Bisulfite PCR, Gel Purification, and Quantification of PCR Products

Predicted CpG islands belonging to the shortlisted stress-responsive genes were PCR amplified for both treated (pesticide and RH) populations and control samples (for details, see ‘Table S3’), using 15 ng of bisulfite-treated genomic DNA as a template. Further, as DNA methylation status can sometimes be under-or over-represented by the underlying naturally occurring variations in DNA sequence, each gene was also PCR amplified from the untreated genomic DNA isolated from individuals of the BOD population. The PCR conditions were different for each of the different genes and were as follows: 2 min initial denaturation at 94 °C, followed by 30 cycles of 30 s at 94 °C, 1 min at 45–62 °C, 1 min at 68–72 °C, and final elongation at 72 °C for 2 min. In general, each PCR reaction (final volume 20–25 μL) consisted of 15 ng bisulfite-treated DNA template, 200 μM dNTPs, ~0.6 U Taq DNA polymerase, 1X Taq buffer, and 13 μM each of forward and reverse primers. Different brands of Taq polymerases were used to amplify the different genes, from the bisulfite converted DNA, i.e., Promega Taq polymerase (Promega, Madison, WI, USA), Kapa 2G Robust (Kapa Biosystems, Roche, MA, USA), Kapa HiFi DNA Polymerase (Kapa Biosystems, Roche, MA, USA), and Bangalore Genei Taq (Bangalore Genei, Bengaluru, India). The PCR amplified products were separated on 1% agarose gel and purified using a nucleic acid extraction kit (Vivantis GF-1 Tissue DNA Extraction Kit). The eluted DNA fragments were quantified using a Qubit 4.0 fluorometer (Invitrogen, MA, USA). The PCR amplicons (corresponding to different genes in treatment) were pooled in amounts and were sent to M/s IMGM Laboratories GmbH, Martinsried, Germany, for library preparation and sequencing.

#### 4.7.4. Library Construction and Illumina-MiSeq Sequencing

DNA libraries were prepared using the NEBNEXT Ultra DNA Library Kit (New England Biolab, Ipswich, MA, USA) using 100 ng DNA as starting material. The library was prepared as per the manufacturer’s protocol and was quantified using the Qubit dsDNA HS (high sensitivity) Assay Kit (Thermo Fisher Scientific, MA, USA). The library pool was denatured using NaOH to produce single-stranded DNA fragments for cluster generation. These fragments were then immobilized onto the flow cell, followed by bridge amplification, resulting in ~1000 copies of the original fragments localized in a tight cluster. Thereafter, sequencing primers were hybridised to the adapter sequences, and bidirectional sequencing was carried out using the MiSeq Reagent Kit v2 500-cycles chemistry on the MiSeq system (Illumina Inc., San Diego, CA, USA). 

#### 4.7.5. Initial Data Processing and Quality Check 

Imaging, data processing, and evaluation of the sequencing run performance were carried out using the Illumina MiSeq Reporter (MSR; v2.5.1.3) and the Illumina Sequence Analysis Viewer (SAV; v 2.4.7). The initial QC and 3′-end adapter trimming were performed using the MiSeq inherited MSR software packages. This was followed by demultiplexing of passed filter reads based on their indices and corresponding sample IDs. The resulting R1 and R2 (.fastq) files, containing quality values and sequence information, were used for downstream analyses. 

#### 4.7.6. Data Analyses

Using a designated pipeline (Figure S2), bisulfite-sequencing reads were filtered, and quality scores were estimated using FastQC [69], followed by adapter trimming using Trim-galore v0.6.5 [70]. Next, the paired-end reads (R1 and R2) were merged using fastq-join [71] and then aligned to the reference (reference genomic DNA sequence of each gene) using BWA-MEM v0.7.17 [72]. The alignment files were imported to SAMTOOLS v1.4 [73] for sorting, demultiplexing, indexing, and estimation of mapping statistics. In addition, to estimate the overall methylation status and coverage calls, the alignment and methylation calling were carried out using bisulfite sequencing aligner software, Bismark v0.20.1 [74]. Post-alignment, an inbuilt bismark_methylation_extractor.pl script was used to extract the methylation call for every single cytosine analysed in all three contexts (CpG, CHG, and CHH). Thereafter, percent methylation in BPH populations, across the selected stress-responsive genes, in all three contexts, was subjected to statistical analyses (i.e., principal component analysis, whisker plots, and hierarchical clustering) using the R package. 

Further, differential methylation analysis was carried out using BiQ Analyser HT [75], with default parameters. The relative methylation scores across samples were determined for each site and in all three contexts (i.e., CpG, CHG, and CHH). Next, the output files (methylation scores) were exported as .csv, and the heatmaps were generated using Heatmapper (http://www.heatmapper.ca/; Accessed on 19 August 2021) for better visualization and interpretation. As the methylation percentage *per se* is not indicative of the actual read coverage of detected methylated or unmethylated reads at a position, the coverage files containing methylation counts for each cytosine and their respective coverage were generated as sorted bedgraph files using BEDTools [76]. These coverage files were used directly as the input for data analysis using Methylkit [77]. Differences in methylation were calculated between stressed and control BPH populations based on Fisher’s exact test. Here, the chi-square test was used as a default setting to calculate the *p*-value, and subsequently, the *p*-value was adjusted to *q*-value using the sliding linear model. After *q*-value calculation, differentially methylated cytosines, with percentage methylation difference ≥25, and *q*-value ≤0.01, were identified. Thereafter, based on DNA methylation values across genes, BPH populations subjected to nutritional stress (RH) and pesticide stress (imidacloprid), along with their respective reversal populations (i.e., after insects were brought back to normal growing conditions), were clustered using Ward’s agglomeration method and Euclidean distance or dissimilarity index, using the custom script provided in the methylkit suite of data analysis programmes. Further, to identify and interpret the influence of life stages and stresses on the methylation status of BPH, pairwise Pearson’s correlation scores were calculated, and scatter plots of percent methylation values were generated across samples using Methylkit. 

### 4.8. DNA Methylation Inhibition Assay

#### 4.8.1. Azacytidine Treatment 

To confirm and validate the functional relevance of DNA methylation for BPH survival, insects (BOD population; adults) were treated with 5-azacytidine (5-aza; an exclusive DNA methylation inhibitor). Thereafter, the effects of 5-aza treatment were evaluated for BPH, viz., its methylation status, and gene expression. For this, BPH adults (~10 insects/tube) were fed on an artificial diet (for details, see [78]), supplemented with 5-aza (40 μM; Sigma-Aldrich, St. Louis, MO, USA), along with phenol red (0.2 μg/μL) as feeding indicator (Figure S3). Phenol red shows a gradual transition from yellow to pink over the pH range of 6.8 to 8.2. This property of phenol red was exploited to ensure the intake of artificial diet (supplemented with 5-aza) by BPH individuals. The artificial media (containing phenol red) had a pH of 7.4 and hence was orange. However, upon ingestion by BPH and passing through its gut (which is alkaline, pH > 8), it caused them to secrete pink honeydew. Hence, secretion of pink-colored honeydew was considered an indication of feeding (Figure S3). The insects were allowed to feed for 48 h, and the artificial diet was replenished after 24 h by transferring the insects to fresh tubes with an artificial diet supplemented with 5-aza and phenol red. Post-treatment, insects were collected for downstream analyses. 

#### 4.8.2. Estimation of DNA Methyltransferase (DNMT) Activity

The inhibitory activity of the 5-aza treatment was validated by the estimation of DNMT activity in the treated insects. For this, the nuclear protein was extracted from the treated and untreated (control) insects using EpiQuik Nuclear Extraction Kit (Epigentek, Farmingdale, NY, USA), following the manufacturer’s instructions. The extracted protein was quantified using the Bradford protein assay kit (Bio-Rad, Hercules, CA, USA). Next, DNMT activity was estimated using 3 μg of the nuclear protein extract for each sample (i.e., treated and untreated) in triplicates, using the EpiQuik DNA Methyltransferase Activity/Inhibition Assay Kit (Epigentek, Farmingdale, NY, USA), as per the manufacturer’s protocol. Pure DNA methyltransferase supplied with the kit was used as a positive control. Methyltransferase activity was calculated using the average absorbance at 450 nm and was expressed as OD/hr/mg. Subsequently, DNMT activity in the treated insects was compared with that of the untreated (control) insects, and a percent reduction in DNMT levels was estimated.

#### 4.8.3. Genomic DNA Extraction from 5-aza Treated Insects

Total genomic DNA was extracted from 5-aza treated and untreated insects using the GF-1 tissue DNA extraction kit (Vivantis, Selangor, Malaysia), following the manufacturer’s instructions and as mentioned earlier. The DNA was quantified using the NanoDrop Spectrophotometer (Thermo Fisher Scientific, MA, USA), and the quality was checked by electrophoresis on 0.8% TBE agarose gel [69]. 

#### 4.8.4. Quantification of Methylation Levels Using Methylation-Sensitive Restriction Digestion Assay

A modified methylation-sensitive restriction assay (MSRA), followed by CpG island amplification-representational difference analysis, was used to confirm whether 5-aza treatment caused any reduction in the methylation of the loci (corresponding to stress-responsive genes) under consideration in this study. For this, methylation-sensitive/insensitive isoschizomeric restriction enzyme pairs, i.e., HpaII/MspI, AatII/FatI, and BsmAI/BstCI (New England Biolabs, Ipswich, MA, USA), were used for digesting the genomic DNA isolated from 5-aza treated and control samples. Each restriction reaction (final volume 25 μL) contained genomic DNA (200 ng), restriction enzyme (2U), buffer (1x), and sterile water (to 25 μL) and was incubated for 6 h at their optimum working temperatures (i.e., 37 °C for HpaII, MspI, and AatII; 55 °C for BsmAI and FatI; 50 °C for BstCI). This was followed by a semiquantitative PCR performed using the same gene-specific primer pairs as listed in Table S7.

PCR reactions were set up for both the digested DNAs (i.e., the one restricted with methylation-sensitive and the other with insensitive restriction enzyme) for each sample. In addition, to normalise for variations in the amounts of input template DNA in the PCR reactions, and to measure the initial amounts of the target of interest, one independent reference (undigested control; UC), was also set up. All PCR reactions (final volume 20 μL) consisted of 200 μM dNTPs, 0.6 U Taq DNA polymerase (Bangalore Genei, Bengaluru, India), 1X Taq buffer, and 13 μM each of forward and reverse primers. PCR was performed using 20 ng of the genomic DNA as a template for digested and undigested reactions. The PCR amplification profile was: initial denaturation at 95 °C for 5 min, followed by 25 cycles of denaturation at 95 °C for the 30s, annealing at 55–60 °C for 30 s, extension at 72 °C for 30 s, and a final extension of 72 °C for 2 min. The PCR amplified products (10 μL) were separated on 1% agarose gel. The relative intensity for each fragment in digested (methylation-sensitive/insensitive restriction digestion) and undigested (control) lanes was quantified using Image Lab software (v6.0.1; Bio-Rad Laboratories, CA, USA). The intensity of the PCR fragment obtained for undigested control (UC; which was not subjected to restriction digestion and, therefore, no reduction in the copy number due to cleavage of the target site) represented 100% methylation. From this, the degree of methylation (defined as the fraction of methylated alleles) for each sample was calculated by dividing the band intensity values obtained for each sample by that obtained for its corresponding PCR fragment amplified from the UC template. 

#### 4.8.5. RNA Extraction

Total RNA was extracted from nine BPH individuals per treatment (i.e., 5-aza, pesticide, and nutritional stress) using RNeasy Plus Mini Kit (Qiagen, Germantown, MD, USA) following the manufacturer’s protocol. The RNA was quantified using the NanoDrop Spectrophotometer, and the quality was checked by gel electrophoresis [68]. 

#### 4.8.6. Gene Expression Analysis

To study the correlation and impact of methylation on gene expression, quantitative real-time PCR (qRT-PCR) was carried out on 5-aza treated insects as well as on BPH insects exposed to stress (nutritional and pesticide) and BOD insects (unexposed to stress) served as control. For this, cDNAs synthesis was performed using the SuperScript IV First-Strand Synthesis System (Invitrogen, NY, USA) using 2.5 μg of the total RNA from each sample. Next, the cDNA was column purified using QIAquick PCR Purification Kit (Qiagen, MD, USA) and then quantified using NanoDrop Spectrophotometer. Quantitative PCR was performed on an Applied Biosystem StepOne Real-Time PCR system (Thermo Fisher Scientific, MA, USA). Each real-time PCR reaction (10 μL) contained 5 μL SYBR Green PCR Master Mix (Thermo Fisher Scientific, MA, USA), 2.5 μM each of forward and reverse primer, and 5 ng of the cDNA samples. Details of all the primer pairs used for qRT-PCR analysis are provided in Table S8. PCR conditions involved initial denaturation at 95 °C for 5 min, followed by 40 cycles of denaturation at 95 °C for 10s and annealing and extension at 60 °C for 30s. After 40 cycles, a melt curve analysis was carried out to determine the specificity of the reaction. Thereafter, the relative gene expression was normalized to the expression level of *Actin* (GenBank accession no.: LOC111057938). The expression level was displayed as a relative expression value based on the relative standard curve method. Results were analysed using the 2^−ΔΔCt^ method built into the StepOnePlus Real-Time PCR analysis software (Applied Biosystems, Waltham, MA, USA) provided with the instrument. All analyses included three biological replicates and three technical replicates. Further, the gene expression and methylation data were subjected to a correlation analysis performed using SPSS Statistics. 

## Data Availability

All data needed to evaluate the conclusions in the paper are presented in the paper and/or the Supplementary Materials. NGS data sets generated during this study are available as Sequence Read Archive (SRA) files at NCBI and can be accessed using the accession number PRJNA783726.

## Supplementary Materials

The following are available online at https://www.mdpi.com/article/10.3390/ijms23158728/s1.
