# Supplementary material for: Heritable Epigenomic Modifications Influence Stress Resilience and Rapid Adaptations in the Brown Planthopper (Nilaparvata lugens)
Source: Int J Mol Sci. 2022 Aug 5;23(15):8728. doi: 10.3390/ijms23158728 (PMC9368798; doi:10.3390/ijms23158728)
Supplement: Supplementary file 1 [file ijms-23-08728-s001.zip › Fig_S1.pdf]

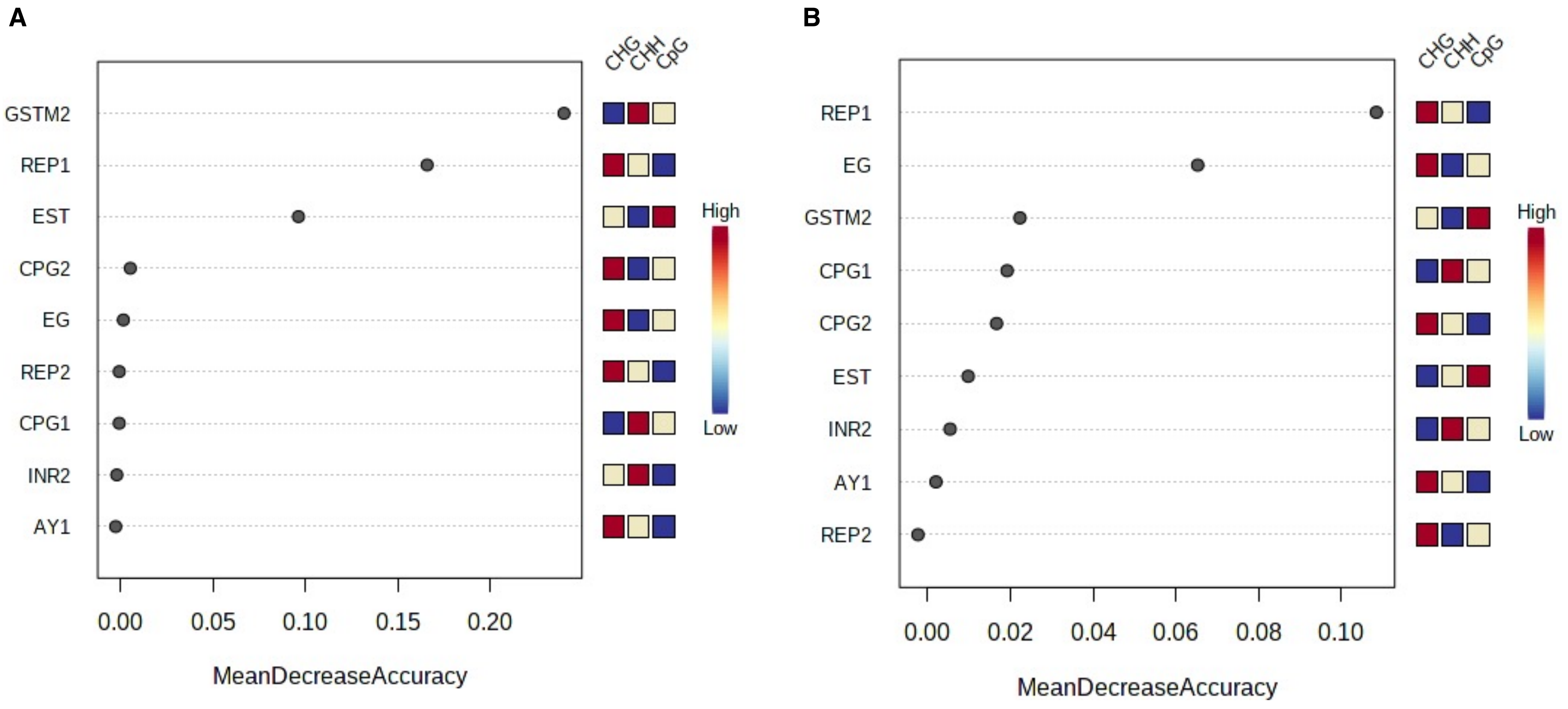


Fig. S1 Random Forest-based Classification indicating the most important variables (genes). The genes are ranked by the mean decrease in classification accuracy when they are permuted. (A) For pesticide-exposed BPH populations (B) For BPH populations fed on RH.
