# Supplementary material for: Heritable Epigenomic Modifications Influence Stress Resilience and Rapid Adaptations in the Brown Planthopper (Nilaparvata lugens)
Source: Int J Mol Sci. 2022 Aug 5;23(15):8728. doi: 10.3390/ijms23158728 (PMC9368798; doi:10.3390/ijms23158728)
Supplement: Supplementary file 1 [file ijms-23-08728-s001.zip › Fig_S2.pdf]

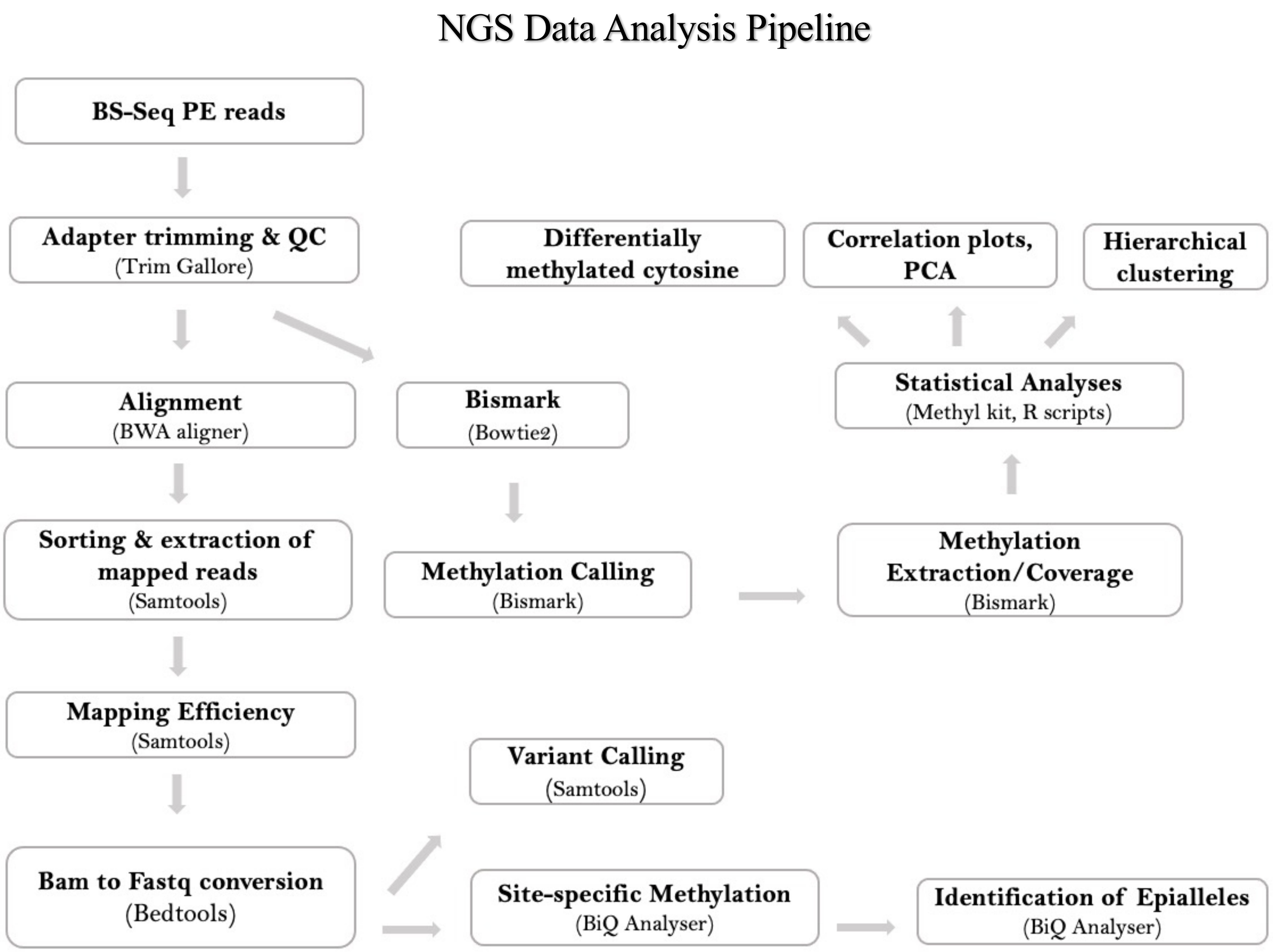

Fig. S2 Pipeline followed for analysing NGS data sets generated during this study.
