# Supplementary material for: Heritable Epigenomic Modifications Influence Stress Resilience and Rapid Adaptations in the Brown Planthopper (Nilaparvata lugens)
Source: Int J Mol Sci. 2022 Aug 5;23(15):8728. doi: 10.3390/ijms23158728 (PMC9368798; doi:10.3390/ijms23158728)
Supplement: Supplementary file 1 [file ijms-23-08728-s001.zip › Fig_S3.pdf]

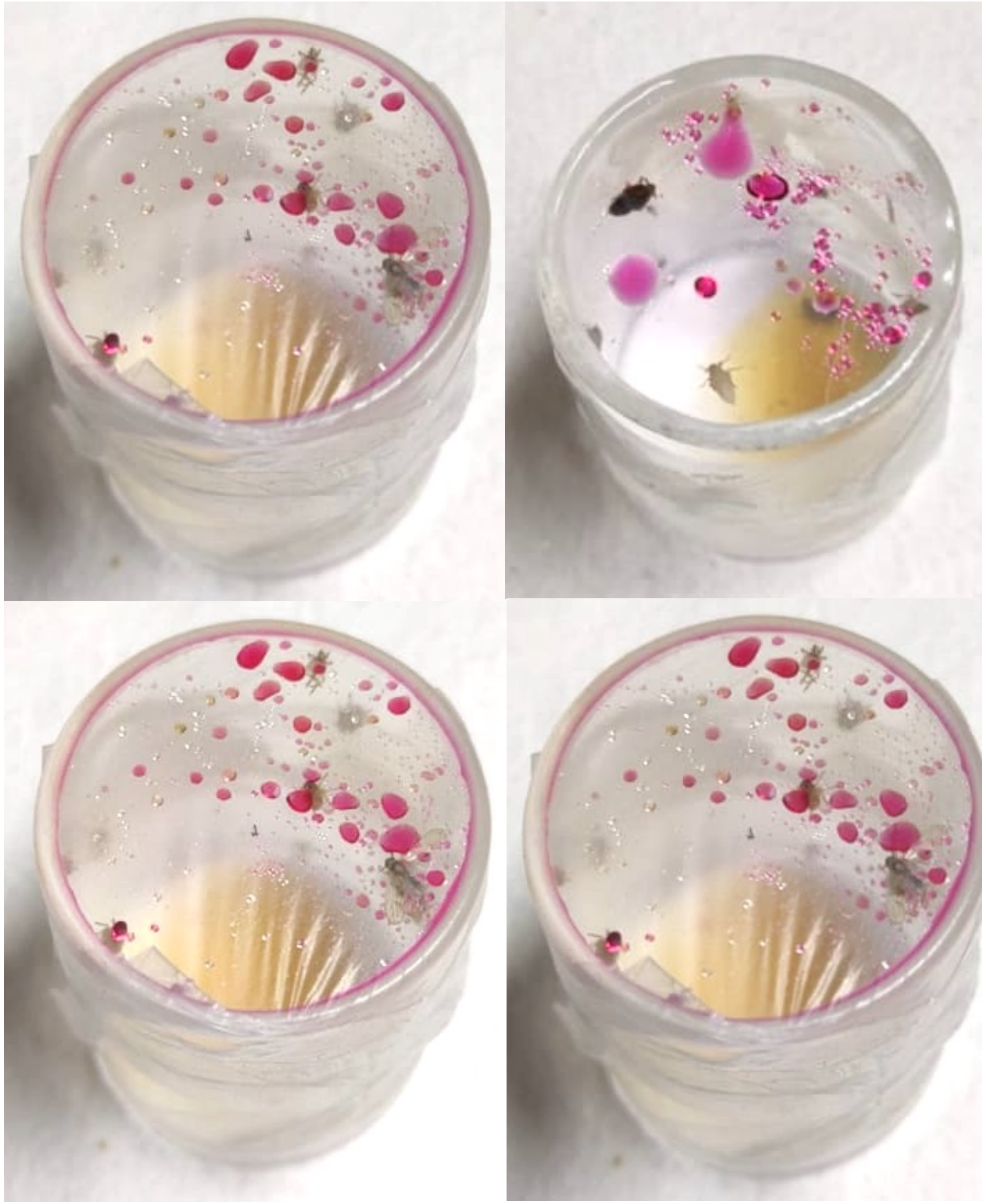

Fig. S3 5-Azacytidine treatment of BPH individuals. BPH feeding on artificial diet (see 'Methods' for details) supplemented with 5-azacytidine and phenol red. The insects were reared in glass cylinders sealed at both ends by double layer of Parafilm. The artificial diet was placed between the two layers of Parafilm at both ends. The pink droplets are honeydew secreted by BPH individuals feeding on the artificial diet. Phenol red exhibits pink colour in alkaline pH and after passage through the insect gut which is alkaline. Pink honeydew confirmed insect feeding on the artificial diet supplemented with 5-azacytidine and phenol red.
