# Supplementary material for: Heritable Epigenomic Modifications Influence Stress Resilience and Rapid Adaptations in the Brown Planthopper (Nilaparvata lugens)
Source: Int J Mol Sci. 2022 Aug 5;23(15):8728. doi: 10.3390/ijms23158728 (PMC9368798; doi:10.3390/ijms23158728)
Supplement: Supplementary file 1 [file ijms-23-08728-s001.zip › Supplementary Tables S1 to S4, S6 to S8.pdf]

Table S1 Stress-responsive genes selected for this study, their role and significance for BPH.

Genes	NCBI accession	Functional relevance for BPH	Reference
Carboxylesterase	LOC111062288	Involved in xenobiotic detoxification	(69)
CYP6AY1	LOC111048315	P450 monooxygenase involved in pesticide degradation	(70)
CYP6ER1	LOC111057329	P450 monooxygenase involved in imidacloprid (pesticide) metabolism	(70, 71)
Gstm2	LOC111061700	Involved in xenobiotic detoxification and host adaptation	(72)
InR2	LOC111047475	Regulates wing polyphenisms in BPH response to environmental cues such as population density and host quality	(49)
Endoglucanase	LOC111056901	Plant cell wall degrading enzyme; facilitates BPH feeding on rice host	(73)

Table S2 Predicted CpG islands along with their respective amplicon lengths and location.

**Genes**	**CpG island (Amplicon) length**	**Location of the CpG island**
Carboxylesterase	278 bp	Exonic
CYP6AY1	297 bp	Exonic
CYP6ER1-CpG1	299 bp	Exonic
CYP6ER1-CpG2	293 bp	Exonic
Gstm2	226 bp	Exonic
InR2	337 bp	Intronic
Endoglucanase	300 bp	Intronic

Table S3 Description of BPH populations analyzed in the present study.

Samples	Description
BOD_AD	BOD adults (Control)
BOD_NY	BOD nymphs (Control)
PI	Parental imidacloprid
IG1	1st generation BPH adults under Imidacloprid stress
IG4	4th generation BPH adults under Imidacloprid stress
ING1	1st generation BPH nymphs under Imidacloprid stress
ING4	4th generation BPH nymphs under Imidacloprid stress
PR	Parental Rathu Heenati (RH)
RG1	1st generation BPH adults feeding on RH plants
RG4	4th generation BPH adults feeding on RH plants
RNG1	1st generation BPH nymphs feeding on RH plants
RNG4	4th generation BPH nymphs feeding on RH plants
IR1	1st generation reversal population after Imidacloprid withdrawal
IR2	2nd generation reversal population after Imidacloprid withdrawal
RT1	1st generation reversal population after RH exposure
RT2	2nd generation reversal population after RH exposure
UTUE	Untreated and Unexposed to stress (Control)

Table S4 Total reads generated on Illumina Miseq System and their mapping efficiency

Samples	Total reads	Filtered reads	>Q30 reads	Mapped reads	Mapping efficiency
**BOD_AD**	551992	551166	99.85036015	475274	86.23064558
**BOD_NY**	544646	438826	80.57086621	348394	79.3922876
**PR**	556836	408648	73.3874965	179630	43.95714649
**RG1**	679769	581712	85.57495267	520887	89.54379487
**RG4**	590407	571952	96.87419018	399789	69.89904747
**RNG1**	550493	546696	99.31025463	538704	98.53812722
**RNG4**	673390	594782	88.32652698	544694	91.57876331
**RT1**	634404	574902	90.62080315	436858	75.98825539
**RT2**	624645	574576	91.98440714	546889	95.18131631
**PI**	431102	427436	99.1496212	137644	32.20224782
**IG1**	680448	556398	81.76936371	410873	73.8451612
**IG4**	544224	513544	94.36261539	453702	88.3472497
**ING1**	509262	433198	85.06387675	386090	89.1255269
**ING4**	618125	590592	95.54572295	385532	65.27890659
**IR1**	592018	546289	92.27574162	167694	30.6969388
**IR2**	326286	326186	99.96935204	81202	24.88675579

Table S5 Percent methylation of genes across samples in all three contexts.

Table S5 is a large table that extends beyond the width of the page and is provided as a separate file in spreadsheet format **(Table S5.xlsx).**

Table S6 Methylation sensitive restriction analysis.

Tested Regions/ Amplicons	BOD1			BOD2			Aza-1			Aza-2		
	S	IS	C	S	IS	C	S	IS	C	S	IS	C
Gstm2	20.6	21.37	100	15.4	17.9	100	11.5	1.2	100	17.17	0.0	100
CYP6ER1	NO SIGNIFICANT CHANGE OBSERVED											
EG	81.6	62.2	100	76	79.5	100	97.1	51.5	100	59.57	63.3	100
CYP6AY1	NA	11.4	100	69.5	NA	100	5	NA	100	NA	NA	100
Est	39.53	34.3	100	100	NA	100	18.0	89.3	100	27.21	19.5	100

NA: Data not available (poor amplification); S: Methylation sensitive restriction enzyme; IS: Methylation insensitive restriction enzyme; C: Control (Uncut)

Table S7 Primers used for bisulfite PCRs performed in this study.

Primer Name	Primer Sequence (5’ – 3’)
CYP6ER1-CpG1 F	GATTATGCATTATTGTGATTACTACA
CYP6ER1-CpG1 R	ACAACGTCTTATAAATTGGTTT
CYP6ER1-CpG2 F	GGCACTGCTACTTCAATCCT
CYP6ER1-CpG2 R	GGAGGTCTCACTAGATTAAAGTTCT
CYP6AY1-F	GAGTGGATGTAACTTTGTTGTAGAG
CYP6AY1-R	TTACTGTCTGGAAGAACRTAGCTATC
EG-F	TGATATTGATGCGATGAGATTAT
EG-R	TTGAAACAACAGGTATTCTTT
Est-1F	TTCGCAAGTTTGGATGATG
Est-1R	CTAACCTCCCTCCATTTCTCT
InR2-F	TGTCGCTGTCGGAGATGGG
InR2-R	CCTCAAAGTTCCAAACAAAATAAT
Gstm2-F	ACCTGAACGACCTTGAAAA
Gstm2-R	AGTACAACTTGCAAGGCCA
Rep1-F	GCCAACTGAACATACCTAC
Rep1-R	ATAAAATGCACACATACCTTA
Rep2-F	TGCTCAGCCCCTCATTCA
Rep2-R	AGGATGACGACACCATTGCT

Table S8 PCR primers for qRT-PCRs.

Primer Name	Primer Sequence (5’ – 3’)
QCYP6AY1 F	GCTGTTTCACTTCTTGAGACTCCG
QCYP6AY1 R	GCTTGAGCTGCTATAACACTCTCTG
QCYP6ER1 F	ATTCCGGTCTATGCGCTTC
QCYP6ER1 R	TGGATTGGCGCTCTCTTACT
QNlIlp2-F	TTCTCAGCCGCTCTAGCAAT
QNlIlp2-R	CAGACGAAGGATCAGGGAAG
QNlEG1 F	ATGATAGAGGGCTGAAGGGTG
QNlEG1 R	TAGTAATGTCGTGGTGCTGGC
QEst mod F	GATTCCTCGGGTTCGCAAGT
QEst mod R	ACCTTGACTTTGCGGCGATAG
